# Effect of disconnection of deformable units on the mobility and stiffness of 3D prismatic modular origami structures using angular kinematics

**DOI:** 10.1038/s41598-021-97609-5

**Published:** 2021-09-14

**Authors:** Kai Xiao, Xiang Zhou, Jaehyung Ju

**Affiliations:** 1 UM-SJTU Joint Institute, Shanghai Jiao Tong University, 800 Dongchuan Road, Shanghai, China; 2 School of Aeronautic and Astronautic Engineering, Shanghai Jiao Tong University, 800 Dongchuan Road, Shanghai, China

**Keywords:** Mechanical engineering, Engineering, Materials science, Mathematics and computing

## Abstract

Architected modular origami structures show potential for future robotic matter owing to their reconfigurability with multiple mobilities. Similar to modular robots, the units of modular origami structures do not need to be assembled in a fully packed fashion; in fact, disconnection can provide more freedom for the design of mobility and functionality. Despite the potential of expanded design freedom, the effect of the disconnection of units on the mobility and physical properties has not yet been explored in modular origami structures. Determining the mobility and weak spots of modular origami structures is significant to enable transformation with minimum energy. Herein, we investigate the effect of the disconnection of units on the mobility and stiffness of architected modular origami structures with deformable units using angular kinematics of geometry and topology of units and closed loops. Angular kinematics provides a valuable tool for investigating the complex mobility of architected modular origami structures with the disconnection of loops. The mobility of the network structure is a function not only of the number of disconnections but also of the topology of the loop. In contrast to the conventional negative perception of defects or disconnection in these materials, the disconnection can potentially be used to expand the design space of mobility for future robotic matter. Our findings can be used to develop powerful design guidelines for topologically reconfigurable structures for soft modular robots, active architected materials, implanted modular devices, deployable structures, thermal metamaterials, and active acoustic metamaterials.

## Introduction

Next-generation artificial materials require exceptional multi-functionality with reconfigurability connected with various sensors and actuators; we refer to these materials as "robotic matter”. Robotic matter that can self-assemble, self-disassemble, and transform its modules to maximize physical performance shows potential for exotic applications such as soft modular robots^1^, active architected materials^2,3^, injected medical implants^4^, shape-morphing propulsion devices^5^, deployable structures^6^, active acoustic metamaterials^7^, and thermal metamaterials^8^.

A design strategy for robotic matter might be found in the features of modular robots as they both involve the assembly of individual units for functionality^9–11^. Modular robots consisting of multiple units assemble and disassemble for 3D motion while maintaining automatic disconnection and rearrangement among units. Notably, the units of modular robots do not all need to be fully packed to facilitate their mobility^9–11^. The module-based scheme has been explored for materials design—customizing physical properties such as Poisson's ratio and directional stiffness by assembling modules^12,13^. However, the modules of functional materials do not possess transformability, limiting their reconfigurability and tunability.

The advanced mobility of modular robots with loose connection among modules can be applied to the design of intelligent architected materials. Among various architected materials, modular origami structures are strong candidates for future robotic matter owing to their versatile transformability—multiple degrees of freedom (DOF) in motion, change of their shape for extreme anisotropic physical properties using a modular function), disconnection, and rearrangement. Unlike the traditional two-dimensional (2D) lattice motion structures^14–16^ and Miura-ori type structures^17–20^, whose transformation patterns have only a single DOF in motion, 3D modular origami structures can possess multi-DOF, enabling dexterous transformability and functionality^21–25^. Advanced modular origami has deformable units and assembles while creating connecting units^18–23^.

One group pioneered the synthesis of 3D reconfigurable modular origami structures with multi-DOF by connecting deformable units^23,24^. Their prismatically deformable 3D building blocks constructed by polyhedral templates showed remarkable tunability^23^ with potential application for tubular acoustic metamaterials^25^. Other groups have suggested different modular units with inverse design^21^ and kinematic analysis of rigid units^22,26^. Similar to modular robots, modular origami units do not need to connect fully during rearrangement. Indeed, the loose connectivity can provide more flexibility to the design of the mobility and functionality. Despite the potential of 3D modular origami for more flexible mobility for robotic matter, however, the effect of the disconnection of units on the overall transposability during the assembly has not yet been explored.

Unlike modular robots whose units are rigid bodies, modular architected material units are generally flexible, resulting in more complex mobility. Moreover, in modular materials, the deformable individual units create additional interconnection with adjacent units, leading to even more complex mobility. Although it is challenging to calculate mobility, the increased mobility can produce an exciting opportunity to realize more versatile reconfigurability of modular architected metamaterials. Therefore, in this work, we suggest a method to determine the mobility and stiffness of architected materials with complex networks connected and disconnected with individual deformable units.

Briefly reviewing a multi-DOF modular origami, we identify independent angles to easily capture individual units’ mobility and interaction with loops in section “Kinematics of a unit cell with multi-DOF”. After investigating the mobility analysis of extended unit cells of cubic modular origami structures produced by planar and spatial tessellations of units in sections “Planar tessellation” and “Spatial tessellation”, we extend the analysis to network structures in section “Mobility analysis of network structures”. We also discuss a directional stiffness of the modular origami to find an optimum direction of mechanical actuation in section “Directional stiffness”. Finally, we conclude our work with significant findings and envision the potential use of our methods.

## Kinematics of a unit cell with multi-DOF

Overvelde et al. synthesized prismatically deformable 3D building blocks with thin-walled structures using "Snapology"; these structures were constructed by extruding the edges of the polyhedron in the normal direction for highly tunable functionality with multi-DOF^23,24^. In cubic-Snapology, the unit cell of the building blocks consists of six tetragons extruded from the cube’s edges in the direction normal to each face^23^. The extruded faces are rigid, yet the whole structures are foldable by the rotational motion of hinges along the edges of the faces. Analogous to a four-bar rotational linkage (4R), each tetragon has a single DOF, coupled with adjacent tetragons to make a cubic-Snapology structure with three DOF^23^. Owing to the folding flexibility arising from the extruded tetragons with 4R, the cubic-Snapology structure possesses superior transformability compared with other polyhedral-based Snapology structures. Therefore, in this work, we confined our interest to cubic-Snapology structures while focusing on their motion when the units are disconnected from adjacent ones.

**Figure 1:**
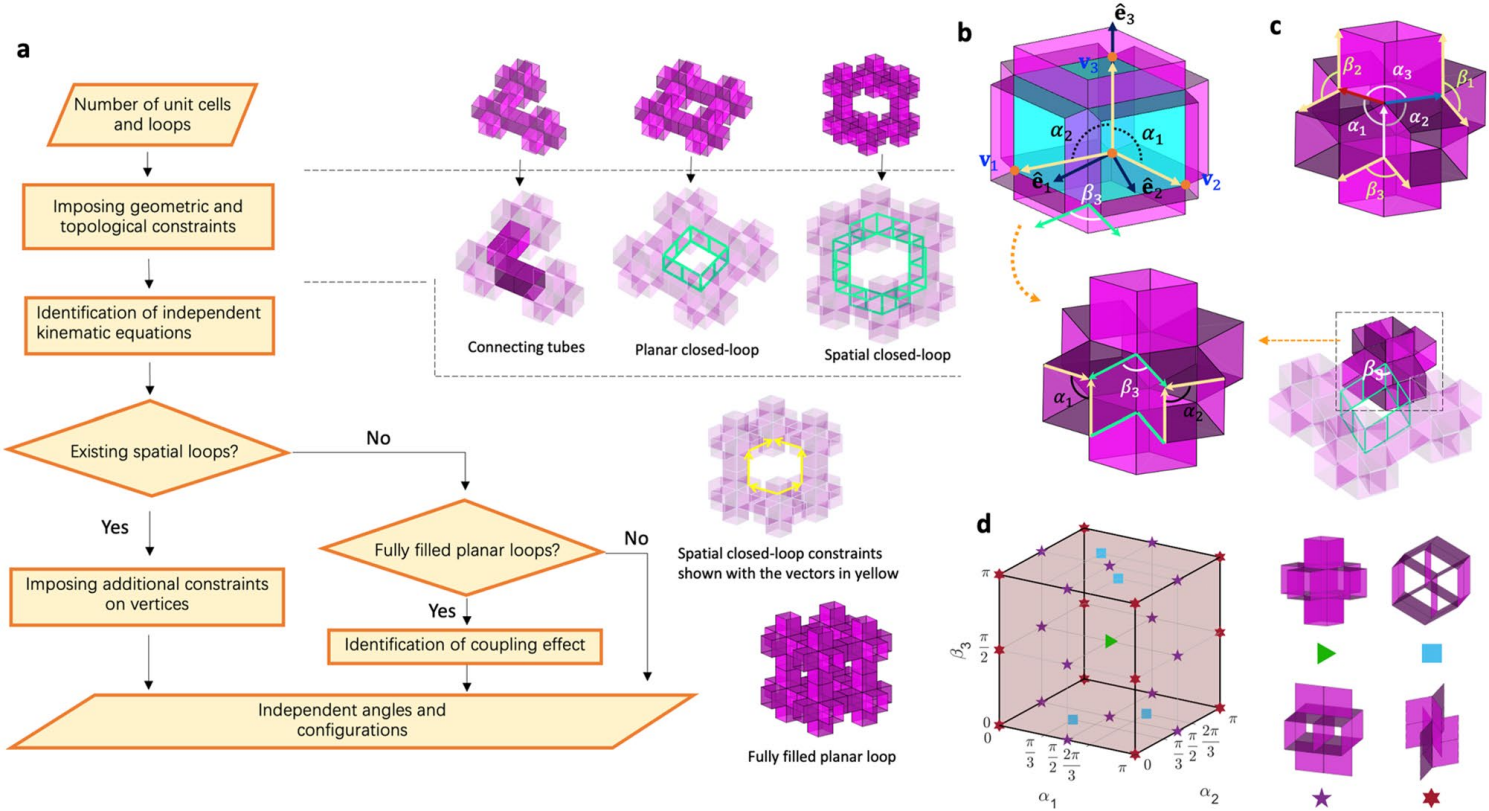
(**a**) Procedure to obtain the independent angles of modular origami structures with disconnection; (**b**) geometric parameters of a prismatic deformable cubic unit; the basis vectors $\hat{\user2{e}}_{1} , \;\hat{\user2{e}}_{2} , \;\hat{\user2{e}}_{3}$ are depicted in the rhombohedron core for two $\alpha$ angles, $\alpha_{1}$ and $\alpha_{2}$. $\beta_{3}$ denotes the angle between adjacent tubular edges, as indicated by the green arrows; (**c**) six angles relevant to the core edges ($\alpha_{1} ,\; \alpha_{2} , \;\alpha_{3}$) and the prismatic tubular edges ($\beta_{1}$, $\beta_{2}$, $\beta_{3}$), (**d**) 3D domain of $\alpha_{1} , \;\alpha_{2}$, and $\beta_{3}$ to represent transformation states of the cubic unit (Supplementary Video 1).

Figure 1a presents an overall strategy to identify the mobility of modular origami structures with cubic units. After counting the total units and loops constructed by units, we impose geometric and topological constraints. These constraints help to sort the units using independent kinematic equations. For spatial loops and filled planar loops, we also impose constraints on the vertices, even reducing the independent kinematic equations. We describe the procedure in sections “Planar tessellation” and “Spatial tessellation” in more detail.

We briefly revisit the kinematics of the deformable cubic unit, a basic layout to analyze tessellated structures while further considering loop connection. Unlike the previous approach for analyzing the kinematics of a cubic-Snapology unit^23^, we use an alternative method to analyze the mobility of the unit by setting two separate angle sets for an internal loop ($\alpha_{1}$, $\alpha_{2}$) and an external loop $\beta_{3}$ ($\in \left[ {0,\pi } \right]$), as illustrated in Fig. 1b. The kinematic analysis with these separate angles can be helpful in identifying the mobility of complex modular assemblies by decomposing the deformation of units and the connection of loops. As a key parameter to conveniently analyze the mobility of a loop connection of units, we use $\beta_{3}$ (the angle between adjacent extruded square tubes), as shown in Fig. 1b. We discuss the kinematics of the loop connection in the next section in more detail.

For a set of orthonormal basis vectors ${\hat{\mathbf{e}}}_{i}$ ($i = 1,{ }2,{\text{ and }}3$) in Fig. 1b, the position vectors ${\mathbf{v}}_{1}$, ${\mathbf{v}}_{2}$, and ${\mathbf{v}}_{3}$ on the edges of an internal rhombohedron core of the cubic unit are expressed as three angles $\alpha_{1}$, $\alpha_{2}$, and $\beta_{3}$:

1 $$ {\mathbf{v}}_{1} = L\sin \alpha_{2} \sin \beta_{3}\, {\hat{\mathbf{e}}}_{1} - L\sin \alpha_{2} \cos \beta_{3}\, {\hat{\mathbf{e}}}_{2} + L\cos \alpha_{2}\, {\hat{\mathbf{e}}}_{3} $$

2 $$ {\mathbf{v}}_{2} = L\sin \alpha_{1}\, {\hat{\mathbf{e}}}_{2} + L\cos \alpha_{1} \, {\hat{\mathbf{e}}}_{3} $$

3 $$ {\mathbf{v}}_{3} = L{\hat{\mathbf{e}}}_{3} $$

As there is no additional constraint imposed by Eqs. (1)–(3), $\alpha_{1}$, $\alpha_{2}$, and $\beta_{3}$ are the independent angles to control the transformation of a cubic modular unit; $0 \le \alpha_{1} ,{ }\alpha_{2} ,\beta_{3} \le \pi$. Therefore, we know that the mobility of a cubic-Snapology unit is three.

Observing the angles on the edges of the cubic unit, we find six angles: three angles relevant to the core edges ($\alpha_{1} ,{ }\alpha_{2} , \alpha_{3}$) and another three angles from the prismatic tubular edges ($\beta_{1}$, $\beta_{2}$, $\beta_{3}$), as shown in Fig. 1c. Alternatively, we can choose another set of independent angles, e.g., $\alpha_{3} = \cos^{ - 1} \frac{{{\mathbf{v}}_{1} \cdot {\mathbf{v}}_{2} }}{{\left| {{\mathbf{v}}_{1} } \right| \cdot \left| {{\mathbf{v}}_{2} } \right|}}$, $\beta_{1} = \cos^{ - 1} \left( {\frac{{\cos \alpha_{2} \cos \alpha_{3} - \cos \alpha_{1} }}{{\sin \alpha_{2} \sin \alpha_{3} }}} \right)$, and $\beta_{2} = \cos^{ - 1} \left( {\frac{{\cos \alpha_{3} \cos \alpha_{1} - \cos \alpha_{2} }}{{\sin \alpha_{3} \sin \alpha_{1} }}} \right)$. The selection of three angles from the six $(\alpha_{1} ,{ }\alpha_{2} , \alpha_{3}$, $\beta_{1}$, $\beta_{2}$, and $\beta_{3}$) provides flexibility when we analyze the complex network structures with loops in the planar and spatial tessellation. Note that the shape of the transformation domain varies depending on the selection of the three independent angles. For example, a combination of $\alpha$ and $\beta$ provides a cubic shape, as shown in Fig. 1d, unlike the core-angle parameterization whose domain is confined in a tetrahedron geometry^23^.

## Planar tessellation

**Figure 2:**
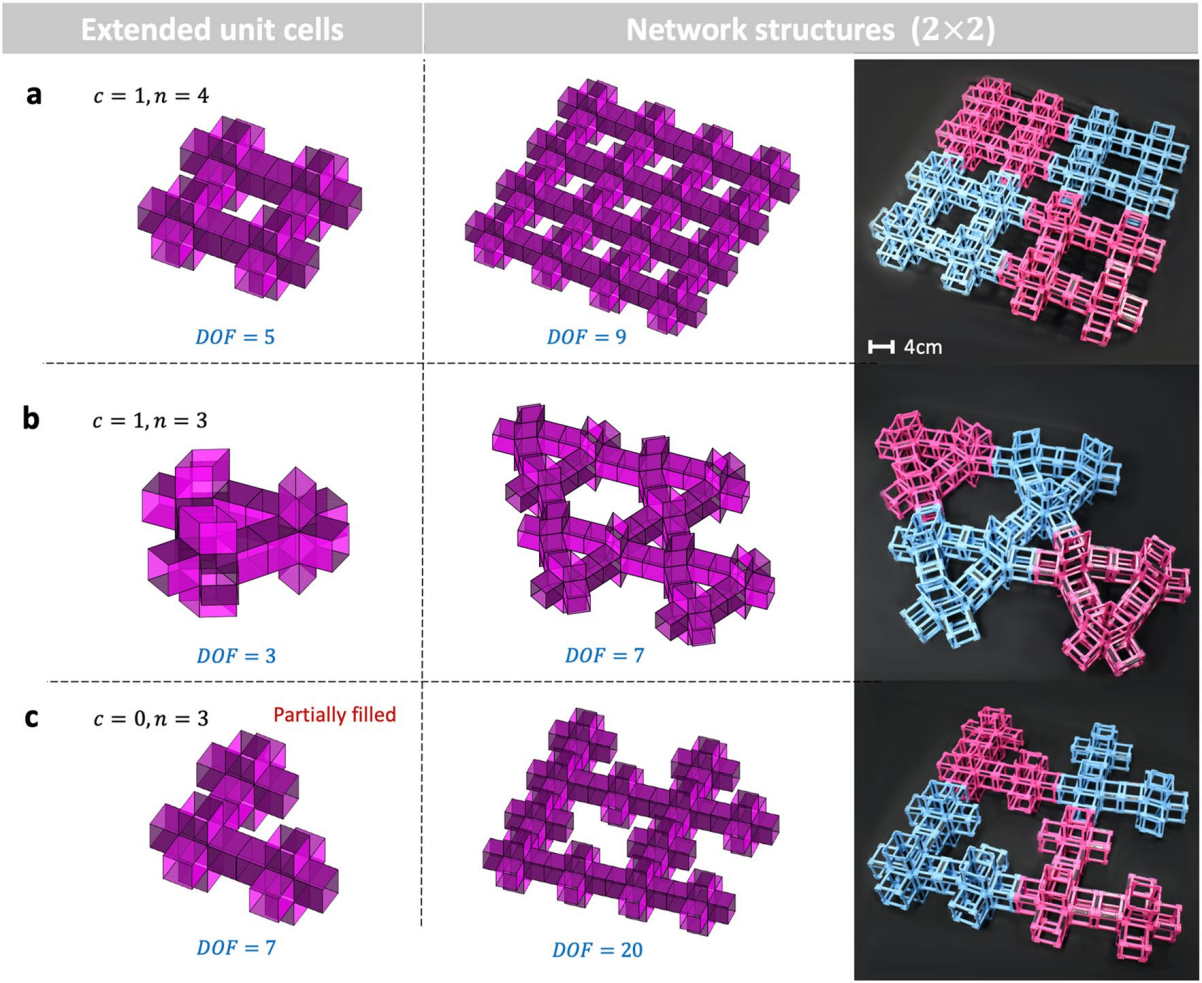
2D extended unit cells and network structures for $3 \le n \le 4$: (**a**) square closed-loop, (**b**) triangular closed-loop, and (**c**) no closed-loop structures; we describe the fabrication method of the prototypes in Section I of the Supplementary Information (SI). The MATLAB code for the computation of mobility of network structures is available in the SI.

To investigate the effect of the disconnection on the mobility of the modular origami network structures, we construct extended unit cells, searching for possible topologies while subtracting units. We need at least four cubic units to build a foldable closed-loop on a planar tessellation: $n = 4$ ($= 2 \times 2$), as shown in Fig. 2a. Subtracting one unit from the extended unit cell with a square loop can generate two possible topologies—one with a triangular loop and the other with no loop—as shown in Fig. 2b,c, respectively. Notably, subtracting two units from the extended unit cell with a square loop makes $n = 2 \times 1$, eventually making an equivalent structure of $n = 4$ ($= 2 \times 2$) after planar tessellation, as shown in Fig. 2a. Therefore, $n = 3$ and $n = 4$ are the only topological options that we can create as extended unit cells for a given maximum $2 \times 2$ unit size.

**Figure 3:**
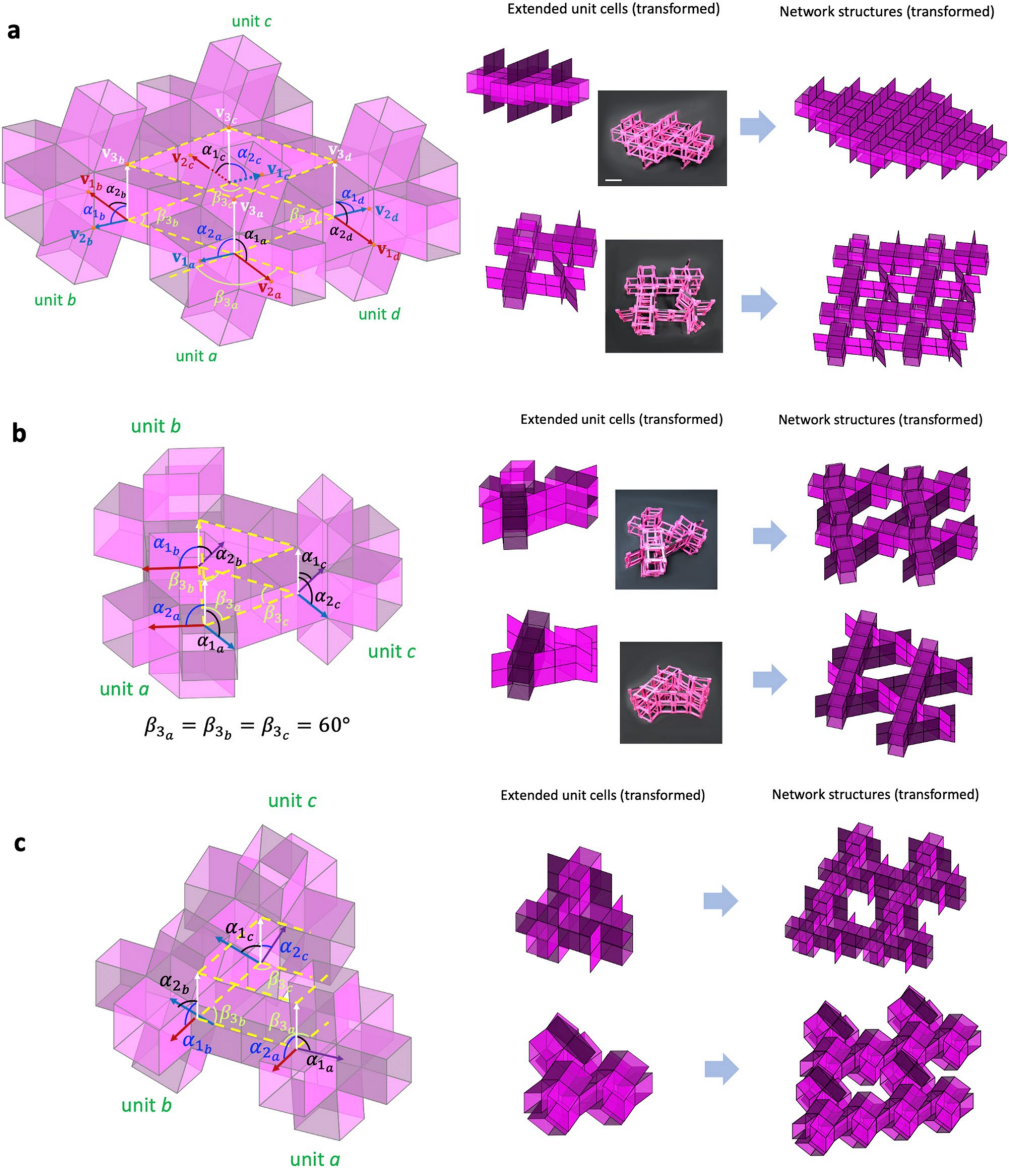
Angular kinematics of motion of 2D extended unit cells and selective transformations: (**a**) square closed-loop (Supplementary Video 2), (**b**) triangular closed-loop, and (**c**) no-loop structures.

The planar tessellation of the cubic units with or without disconnection generates a loop, providing unique mobility. Figure 3 helps us determine the mobility of the extended unit cells with loops. The extended unit cell with a square closed-loop consists of four units. Each unit has three DOF, providing 12 DOF ($= 3 \times 4$) for the connected structure with four units without considering a loop. However, the construction of a loop in Fig. 3a generates constraints of motion at the four tubular junctions of the units:

4 $$ \alpha_{{1_{b} }} = \alpha_{{2_{a} }} ; \alpha_{{1_{c} }} = \alpha_{{2_{b} }} ;{ }\alpha_{{1_{d} }} = \alpha_{{2_{c} }} ; { }\alpha_{{1_{a} }} = \alpha_{{2_{d} }} .{ } $$

From the observation of the loop in Fig. 3a, ${\mathbf{v}}_{3} { }$ for all the units are in parallel and create a parallelogram, introducing three constraints of $\beta$ for one square closed-loop:

5 $$ { }\beta_{{3_{a} }} = { }\beta_{{3_{c} }} ;{ }\beta_{{3_{a} }} + { }\beta_{{3_{d} }} = \pi ;{ }\beta_{{3_{b} }} = { }\beta_{{3_{d} }} { }. $$

Equations (4) and (5) provide a total of seven independent constraints. Therefore, the mobility of the extended unit cell with a square closed-loop in Fig. 3a has five DOF ($= 12 - 7$), whose five independent angles can be, e.g., $0 \le \alpha_{{1_{a} }}$, $\alpha_{{2_{a} }}$, $\alpha_{{1_{c} }}$, $\alpha_{{2_{c} }} ,{ }\beta_{{3_{a} }} \le \pi$.

Similarly, we can apply the method to the extended unit cell with a triangular closed-loop in Fig. 3b. In this case, the total DOF from three units without considering the loop constraint is nine ($= 3 \times 3$). However, the triangular closed-loop provides the three constraints $\beta_{{3_{a} }} = \beta_{{3_{b} }} = \beta_{{3_{c} }} = 60^\circ$, implying zero mobility on the loop with a constant angle. Notably, three binary links making a triangular shape provide zero DOF^27^. The tubular connection imposes additional constraints on the local units: $\alpha_{{1_{b} }} = \alpha_{{2_{a} }} , \alpha_{{1_{c} }} = \alpha_{{2_{b} }} ,{\text{ and }}\alpha_{{1_{a} }} = \alpha_{{2_{c} }}$, which leads to a total of three DOF for the extended unit cell, as shown in Fig. 3b. The transformation domain is on $0 \le \alpha_{{1_{a} }}$, $\alpha_{{2_{a} }}$, $\alpha_{{1_{c} }} \le \pi$. The extended unit cell in Fig. 3c does not have a closed-loop, implying all the $\beta$s are independent angles. Imposing constraints on the two tubular connections: $\alpha_{{1_{c} }} = \alpha_{{2_{b} }}$ and $\alpha_{{1_{b} }} = \alpha_{{2_{a} }}$, we have seven DOF ($9 - 2$) with a transformation domain of $0 \le \alpha_{{1_{a} }} , \alpha_{{1_{b} }} , \alpha_{{1_{c} }} , \alpha_{{2_{c} }} , \beta_{{3_{a} }} , \beta_{{3_{b} }} ,\beta_{{3_{c} }} \le \pi$.

Identifying the independent angles of the extended unit cells helps generate transformation states, as shown in Fig. 3, where the selected transformation shapes of extended unit cells and their network structures are displayed. Unlike for other origami structures and architected materials^20,21^, the mobility of the modular origami structures in this work depends on both the topology of the loops and the individual units. The deformability of the cubic-Snapology units makes the mobility of the network structures complex and unique.

## Spatial tessellation

We use a 3D fully extended unit cell with $2 \times 2 \times 2$ units as a fundamental building block that can fully deform both the loops and units. However, our approach can be readily extended to a more extensive set of extended unit cells, e.g., $3 \times 3 \times 3$.

**Figure 4:**
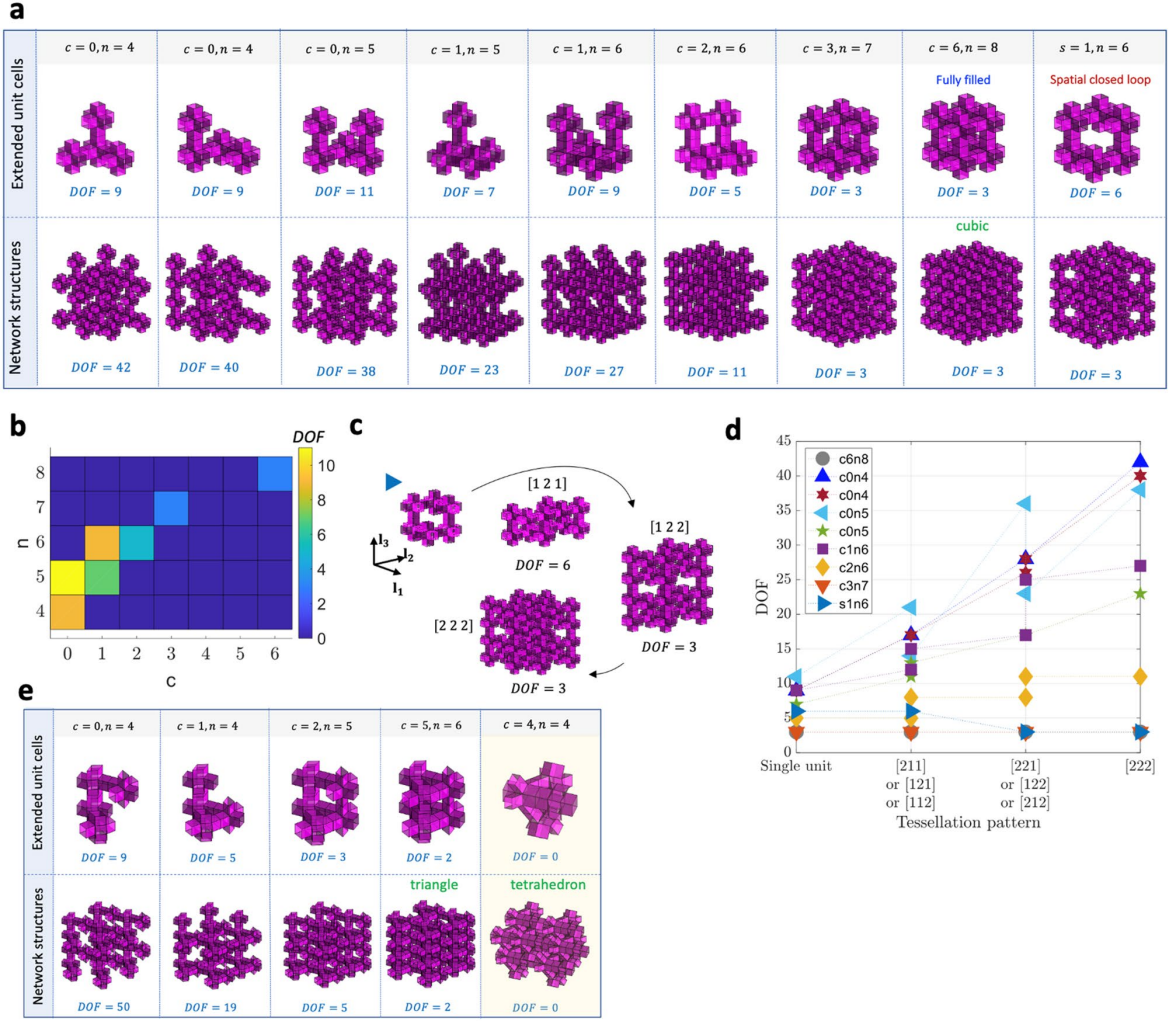
Mobility of 3D extended unit cells and network structures with cubic-Snapology units;$$ n$$ and $$c$$ denote the numbers of units and loops, respectively, and $$s$$ denotes the number of spatial loops. (**a**) Configurations and mobility of extended unit cells and network structures for cubic packing; (**b**) a phase map of mobility of the 3D extended unit cells for varying $$n$$ and $$c$$; (**c**) tessellation of an extended unit cell containing a spatial closed-loop along three lattice vectors $${\varvec{l}}_{1} ,\;{\varvec{l}}_{2}$$, and $${\varvec{l}}_{3}$$. Note that $$\left[ {1 2 1} \right],\;\left[ {1 2 2} \right],$$ and $$\left[ {2 2 2} \right]$$ are the index forms to identify the number of extended units along the lattice vectors. For example, $$\left[ {1 2 1} \right]$$ represents a tessellation pattern with one unit, two units, and one unit along the $${\varvec{l}}_{1} , \;{\varvec{l}}_{2} ,\user2{ }\;{\text{and}}\;{\varvec{l}}_{3}$$ directions, respectively; (d) mobility of cubic assemblies of extended unit cells, where $$X$$ and $$Y$$ in the legend $$cXnY$$ denote the number of loops and units of assemblies, respectively; (**e**) configurations and mobility of extended unit cells and network structures for triangular and tetrahedron packing. The MATLAB code for the computation of mobility of network structures is available in the SI.

Within the scope of the 3D building block, we find three fully filled 3D extended units: cubic, triangular, and tetrahedral shapes, as shown in Fig. 4. We obtain eight independent shapes of partially filled cubic units by subtracting units from the fully filled extended cubic unit cell in Fig. 4a. As observed in Fig. 4a,b, increasing the number of defects (or disconnection) generally increases the mobility of the extended unit cells and network structures. Without a loop ($c = 0$), the mobility increases with $n$ due to the simply added individual units’ motion. However, the increase of $n$ does not always translate into higher mobility. For example, the extended unit cells with $n = 7{ }$ and $8$ have three DOF, lower than that of the extended unit cells with $n < 7$ because of the increased number of loops. Notably, the defect of one unit ($n = 7$) has the same mobility as the filled structure, indicating that one can reduce the mass by $12.5{\text{\% }}$ while retaining control of the architected materials with the same DOF. Interestingly, an extended unit cell with $n = 6$ generates a spatial loop ($s = 1$), providing six DOF. However, its network structure with the spatial loop has only three DOF because of additional constraints during spatial tessellation, providing a $25{\text{\% }}$ reduction of mass with the same mobility as the filled structure.

Conventionally, investigation of the unit cell of architected materials to represent the properties of the whole network is preferred. This is true for architected materials with fully filled units ($c = 6,n = 8,$ referred to as $c6n8$), where the network structures have the same mobility $\left( {DOF = 3} \right)$ as the extended unit cell. However, the assembly with disconnection exhibits a different trend in the mobility compared with the assembly with fully connected units. For example, most extended units with defects ($c0n4$, $c0n5$, $c1n6$, $c2n6$, $c3n7$) increase the mobility if the network size becomes more prominent, which means the overall reconfigurability of the assembly cannot be simplified as the behavior of the unit. However, the mobility of the spatial closed-loop network structure with disconnection ($s1n6$) has the same as that of the cubic unit, not the same as the extended unit cell’s mobility; the mobility of the network structure converges to three from six when the stacked scale increases, as shown in Fig. 4c,d.

In addition, there are three partially filled extended unit cells $\left( {c = 1, 2, 5} \right)$ for the triangular shape, as shown in Fig. 4e. A similar trend is observed in the mobility of the triangular extended unit cells; an increase in $c$ results in a dramatic drop in the mobility of extended unit cells. Interestingly, there is no partially filled extended unit cell for the tetrahedral shape, as shown in Fig. 4e; the fully filled tetrahedral extended unit cell $\left( {c = 4} \right)$ has zero DOF. We analyze the mobility of several extended unit cells with unique features in the following sub-sections.

### Planar closed-loops

#### Extended unit cell with two planar closed-loops

**Figure 5:**
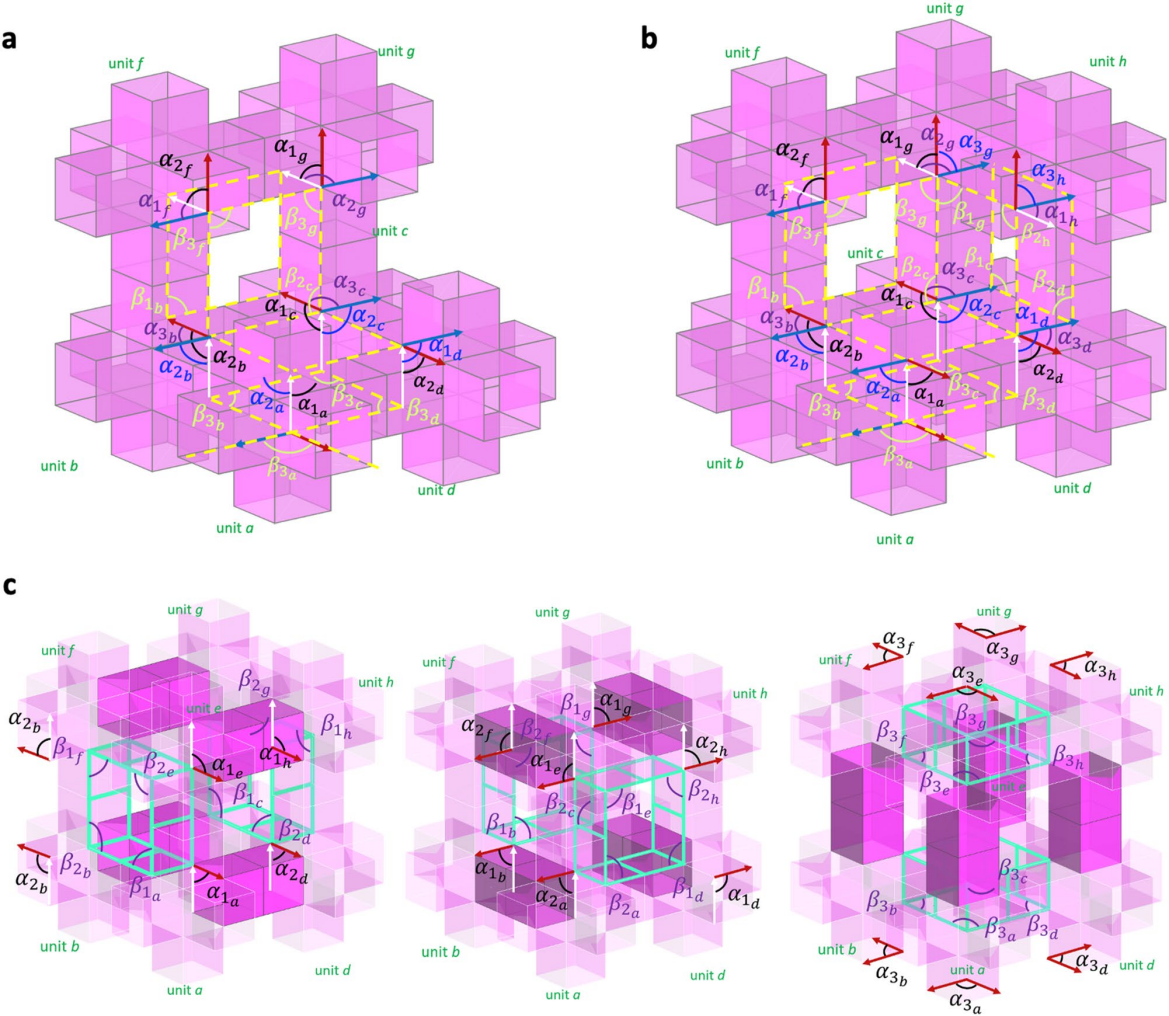
Angular kinematics to determine mobility of extended unit cells with planar closed-loops for spatial tessellation: (**a**) extended unit cell with two planar closed-loops, (**b**) extended unit cell with three planar closed-loops, and (**c**) extended unit cell with six planar closed-loops.

The extended unit cell in Fig. 5a consists of six units. Out of a total of 18 ($= 6 \times 3$) unconstrained conditions with six units, we have the initial independent geometric parameters: $\alpha_{{1_{f} }} ,$ $\alpha_{{2_{f} }} ,$ $\beta_{{3_{f} }} ,$ $\alpha_{{1_{g} }} ,$ $\alpha_{{2_{g} }} ,$ $\beta_{{3_{g} }} ,$ $\beta_{{1_{b} }} ,\alpha_{{2_{b} }} ,$ $\beta_{{3_{b} }} , \beta_{{2_{c} }} ,\alpha_{{1_{c} }} ,$ $\beta_{{3_{c} }} ,$ $\alpha_{{1_{a} }} ,$ $\alpha_{{2_{a} }} ,$ $\beta_{{3_{a} }} ,$ $\alpha_{{1_{d} }} ,$ $\alpha_{{2_{d} }} ,$ and $\beta_{{3_{d} }}$. Two square closed-loops impose six constraints ($= 2 \times 3$): $\beta_{{1_{b} }} = \beta_{{3_{g} }} , \beta_{{1_{b} }} = \pi - \beta_{{3_{f} }} , \beta_{{1_{b} }} = \pi - \beta_{{2_{c} }} , \beta_{{3_{b} }} = \beta_{{3_{d} }} , \beta_{{3_{b} }} = \pi - \beta_{{3_{c} }} , \beta_{{3_{b} }} = \pi - \beta_{{3_{a} }}$. The seven tubular connections require seven constraints: $\alpha_{{2_{f} }} = \alpha_{{1_{g} }} ,$ $\alpha_{{2_{b} }} = \alpha_{{1_{c} }} , \alpha_{{2_{d} }} = \alpha_{{1_{a} }} ,$ $\alpha_{{2_{g} }} = \alpha_{{3_{c} }} ,$ $\alpha_{{1_{f} }} = \alpha_{{3_{b} }} ,$ $\alpha_{{2_{a} }} = \alpha_{{1_{b} }} ,$ and $\alpha_{{1_{d} }} = \alpha_{{2_{c} }}$. Therefore, the extended unit cell in Fig. 5a has five DOF ($= 18 - 6 - 7$) whose transformation is expressed in a domain of $0 < \alpha_{{1_{a} }} , \alpha_{{1_{c} }} , \alpha_{{2_{f} }} , \beta_{{3_{b} }} , \beta_{{1_{b} }} < \pi .$ Notably, the loops perpendicular to each other in Fig. 5a do not affect the individual motions of the adjacent loops. Also note that $\alpha_{{3_{c} }}$, $\alpha_{{3_{b} }}$, $\alpha_{{1_{b} }}$, and $\alpha_{{2_{c} }}$ are functions of $\alpha$ and $\beta$ whose dependencies are expressed in Section II of the Supplementary Information (SI).

#### Extended unit cell with three planar closed-loops

The extended unit cell in Fig. 5b consists of seven cubic-Snapology units with three planar closed-loops, providing a total of 21 ($= 7 \times 3$) DOF if unconstrained: $\alpha_{{1_{f} }} ,$ $\alpha_{{2_{f} }} ,$ $\beta_{{3_{f} }} ,$ $\alpha_{{1_{g} }} ,$ $\alpha_{{2_{g} }} ,$ $\beta_{{3_{g} }} ,$ $\beta_{{1_{b} }} ,\alpha_{{2_{b} }} ,$ $\beta_{{3_{b} }} , \beta_{{2_{c} }} ,\alpha_{{1_{c} }} ,$ $\beta_{{3_{c} }} ,$ $\alpha_{{1_{a} }} ,$ $\alpha_{{2_{a} }} ,$ $\beta_{{3_{a} }} ,$ $\alpha_{{1_{d} }} ,$ $\alpha_{{2_{d} }} ,$ $\beta_{{3_{d} }}$, $\alpha_{{1_{h} }} ,$ $\alpha_{{3_{h} }} ,$ and $\beta_{{2_{h} }}$. However, the three square closed-loop imposes nine ($= 3 \times 3$) constraints and an additional nine constraints on the tubular connections. Therefore, the extended unit cell has three DOF ($= 21 - 9 - 9$) with a possible mobility domain such as $0 < \beta_{{1_{b} }} ,\; \beta_{{3_{b} }} , \;\alpha_{{2_{c} }} < \pi$. Notably, the extended unit cell with three planar closed-loops also does not show a coupling effect of motion among the loops.

#### Extended unit cell with fully filled cubic connection

Unlike the partially filled cases, the extended unit cell with a fully filled cubic connection in Fig. 5c shows a unique feature on the tubular constraints. From the 24 DOF ($= 8 \times 3$) with eight unconstrained units, we subtract constraints considering loops and tubular connections. The extended unit cell has six square closed-loops and 12 tubular connections, providing 30 ($= 6 \times 3 + 12$) constraints, implying an over-constrained condition ($DOF = - 6$). However, the structure in Fig. 5c does have three DOF^23^, showing that our previous method does not apply to this structure.

In this case, we observe a coupling effect of motion on the loops parallel to each other. A parallel loop pair provides one independent constraint. Therefore, there are three independent constraints in the orthogonal directions considering the three parallel loop pairs; e.g., $\beta_{{1_{a} }} = \beta_{{2_{d} }}$, $\beta_{{2_{a} }} = \beta_{{1_{b} }}$, and ${ }\beta_{{3_{e} }} = \beta_{{3_{a} }}$ in Fig. 5c. Therefore, the structure in Fig. 5c has three DOF ($= 24 - 6 \times 3 - 3$). We can express the transformation domain with three angles, $\beta_{{1_{{\text{a}}} }} ,{ }\;\beta_{{2_{a} }} ,{ }$ and $\beta_{{3_{a} }}$; $\beta_{{1_{{\text{a}}} }} + \beta_{{2_{a} }} \ge \beta_{{3_{a} }} ,{ }$ $\beta_{{1_{{\text{a}}} }} + \beta_{{3_{a} }} \ge \beta_{{2_{a} }}$, and $\beta_{{2_{a} }} + \beta_{{3_{a} }} \ge \beta_{{1_{{\text{a}}} }} ,{ }\;\beta_{{1_{{\text{a}}} }} + \beta_{{2_{a} }} + \beta_{{3_{a} }} \le 2\pi .$ Note that the extended unit cells in Fig. 5a,b do not have a parallel loop pair; they only have perpendicular loop pairs. This finding with cubic units strengthens the results in previous work^23^, demonstrating that the cubic-Snapology unit and the extended unit cell with fully filled connection have the same mobility ($DOF = 3$).

### Spatial closed-loops

**Figure 6:**
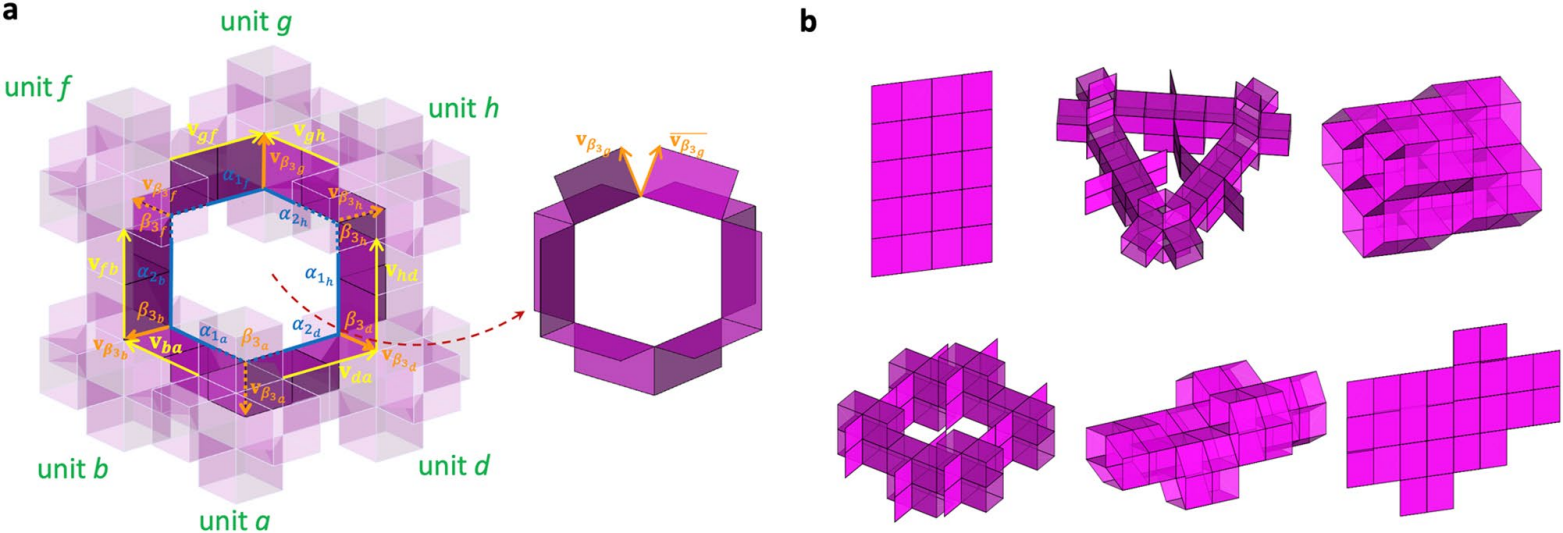
(**a**) Angular kinematics to determine mobility of the extended unit cell with spatial closed-loops and (**b**) its transformed configurations.

Subtracting two units in a spatial diagonal direction from the fully filled extended unit cell with $n = 8$, we can build a spatial closed-loop, as shown in Fig. 6a. If we only consider the mobility of unconstrained six cubic units and six tubular constraints, we have 12 DOF ($= 6 \times 3 - 6$). However, on the tubular connection of the spatial closed-loop, each vertex of the spatial loop imposes additional constraints: the vectors to the exterior directions coincide with each other at the junctions, providing an additional six constraints at the six vertices. Therefore, the extended unit cell with a spatial closed-loop has six DOF ($= 6 \times 3 - 6 - 6$). Figure 6b shows the transformed shapes of the extended unit cells with the spatially closed loop. We provide a more detailed derivation of the mobility of the spatial loop in Section III of the SI.

The exceeding DOF of the extended unit cell with the spatial closed-loop over the fully filled one can transform to various shapes. More interestingly, the network structures constructed by a spatial tessellation of the extended unit cell in Fig. 6a have the same DOF as the network structure with filled units. The matter with the spatial loop reduces the mass compared with the fully filled matter to produce the same DOF ($= 3$) and can produce multiple DOF ($= 3$ or $6$) depending on the modular stage. Identifying the independent motion of modular origami structures with disconnection is significant to reduce unnecessary actuators, eventually resulting in tremendous energy savings for the transformation of robotic matter.

## Mobility analysis of network structures

**Figure 7:**
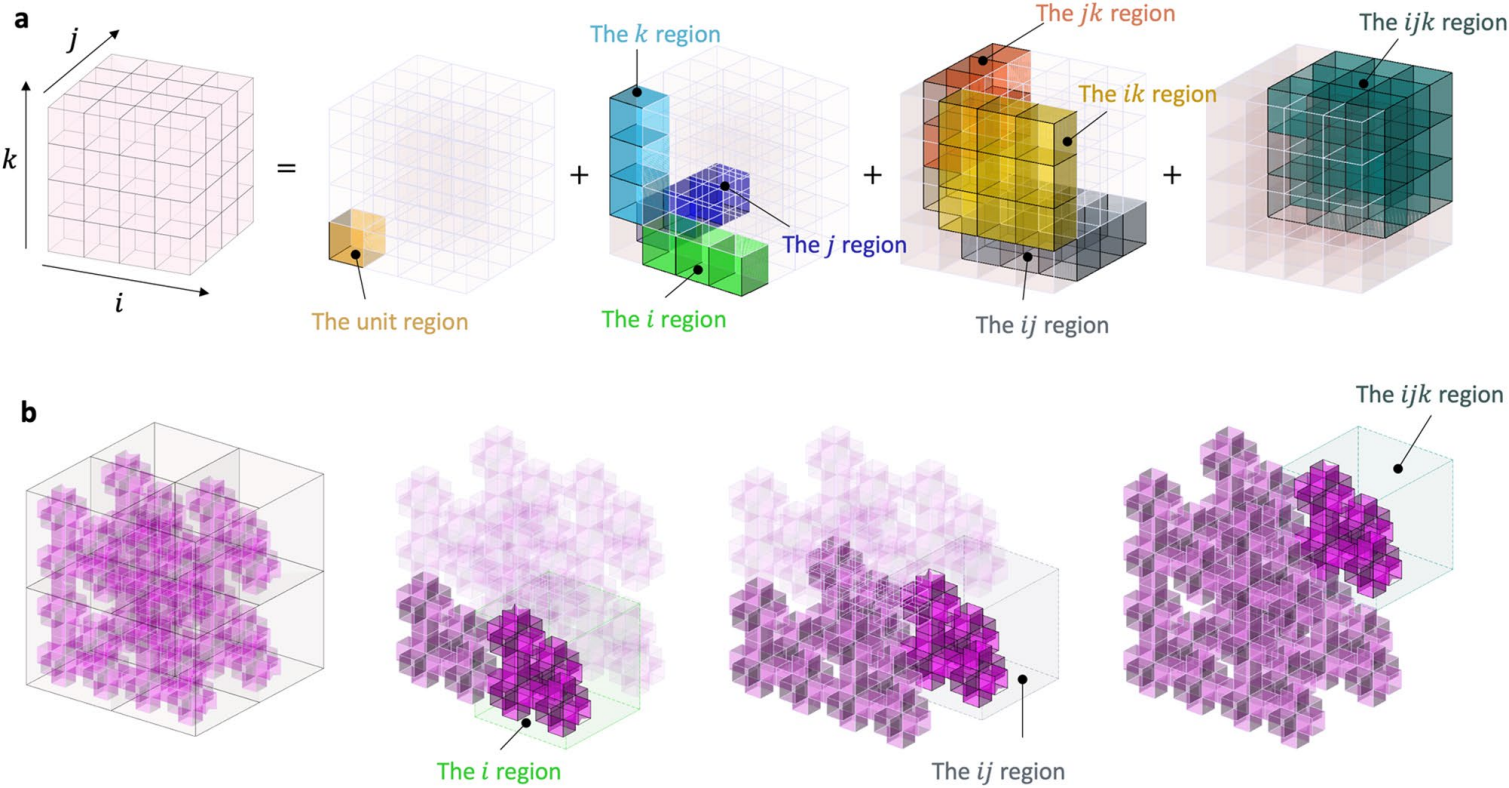
Analytical procedure to obtain mobility of network structures; (**a**) divided regions in a 3D space (**b**) an example of network structure with an extended unit cell (n = 4, c = 0).

Observing the mobility analysis of extended unit cells in the previous sections, we subtract the number of kinematic constraints by the closed-loops and tubular connections from the individual cubic Snapology units’ mobility. Similarly, we can apply the mobility analysis of the extended unit cells to the network structures. However, we need to investigate the mobility in the divided regions of a network structure because each region has a different role in kinematic constraints, as illustrated in Fig. 7a. From the all-possible individual mobility of extended unit cells, we subtract the number of new constraints in each region by the interaction of the extended unit cells with the adjacent regions’ ones, e.g., new planar and spatial loops between extended unit cells and new tubular connections. For example, a network structure with $2 \times 2 \times 2$ extended unit cells $\left( {n = 4, \;c = 0} \right)$ in Fig. 7b has the following mobility for a spatial tessellation:

6 $$ DOF_{n = 4, c = 0}^{S} = 2\left( {i + j + k} \right) + 3\left( {ij + jk + ik} \right) - 6 $$

where the subscript $S$ denotes a spatial tessellation, $i$, $j$, $k$, $ij$, $jk$, and $ik$ are the number of extended unit cells in the $i$, $j$, $k$, $ij$, $jk$, and $ik$ regions. Note that the $ijk$ region provides no independent mobility of the extended unit cell. In detail, we show the derivation of the mobility of network structures for planar and spatial tessellations in Section VI of the SI.

We validate the analytical method with a numerical approach that inspects the rank of the matrices consisting of linearized kinematic constraints^28^. We use the numerical approach to obtain the mobility of network structures with defects where the numerical code for Figs. 2 and 4 are available in the SI. Notably, we implement the same assumption as^23^ that the face of defected origami is rigid, demonstrating that our numerical results of mobility with defects provide the same ones as the eigenmode analysis^23^.

## Directional stiffness

**Figure 8:**
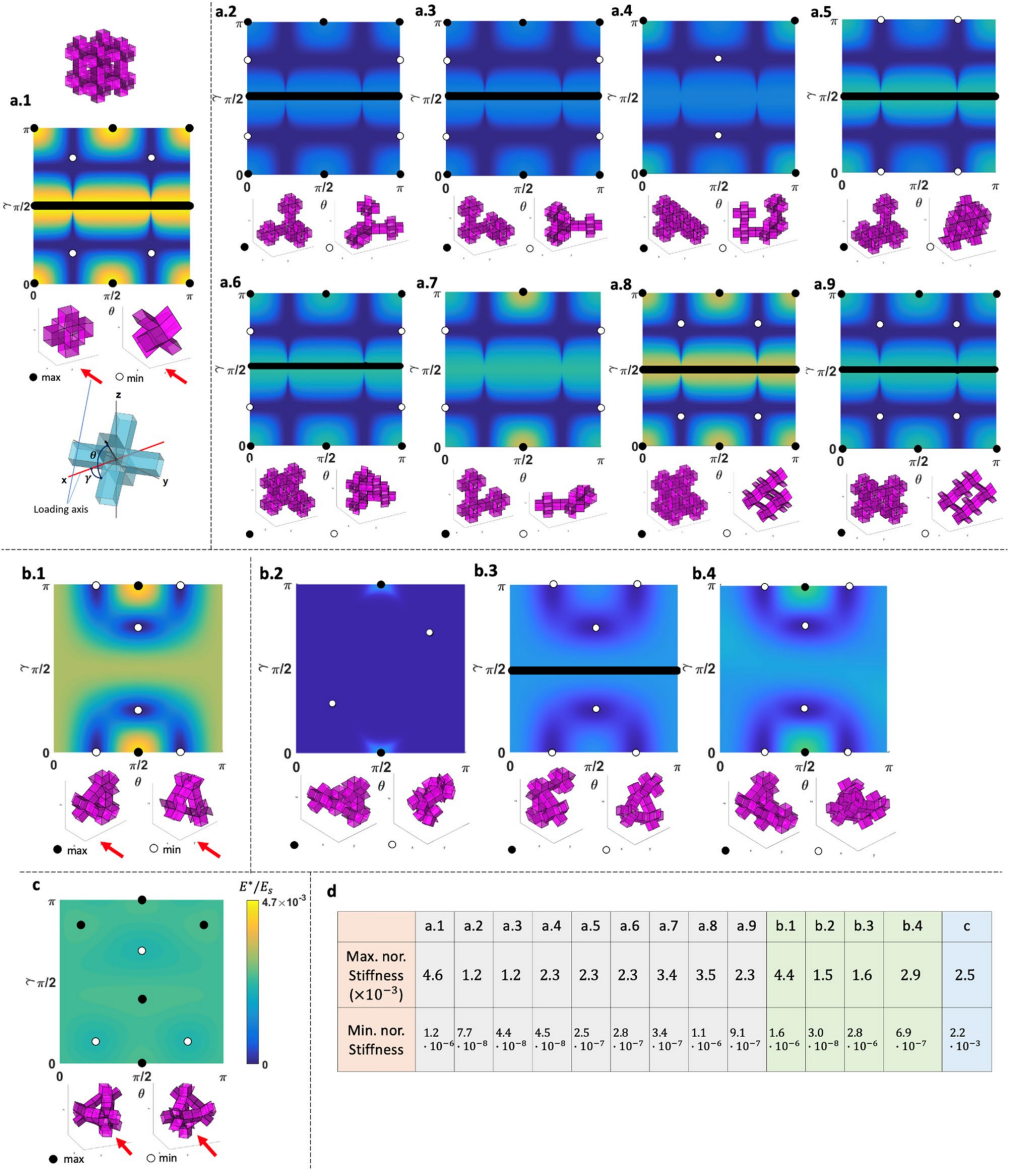
Directional stiffness of modular origami structures for varying loop topologies of (**a**) cubic (Supplementary Video 3), (**b**) triangular, and (**c**) tetrahedron units as a function of angles $\gamma$ and $\theta$. The black and white circles indicate the unit's orientations with maximum and minimum stiffness in each plot. Under each stiffness plot, the deformed units are shown on a magnified scale to facilitate visualization. (**d**) Specific stiffness values with the corresponding number of closed loops $c$, units $n$, and DOF.

The modular origami in this work can be used for both structures and mechanisms. For structural function, it has a load-bearing capacity with direction-dependent stiffness. Once a force exceeds the direction-dependent stiffness, the modular origami starts deforming for transformation—actuation. Unlike the previous study^23^ that used air pressure on the hinges for actuation, one can use a mechanical actuation for reconfigurability^29,30^. Identifying the direction of weak stiffness of transformable modular origami structures with missing connections is significant in search of loading directions to trigger mechanical actuation. Under a small deformation assumption, we obtain the directional stiffness of the modular origami structures with a periodic boundary condition using a finite element (FE)-based discretization method. We implement a torsional spring on the foldable hinge and a set of springs for stretching, bending, and shearing on the deformable faces. See the details on the formulation in Section V of the SI with the MATLAB code. Subtracting units from the fully connected structure can release both the maximum and minimum stiffness peaks, as shown in Fig. 8. The reduced minimum stiffness peak can trigger a transformation, requiring only a tiny amount of energy to trigger motion. For example, Fig. 8a-9 has the same DOF as the structure in Fig. 8a-1. However, a partially filled structure in Fig. 8a-9 has a lower mass and lower energy required to trigger motion than the fully connected structures in Fig. 8a-1.

The triangular loops in Fig. 8b have in-plane isotropy, showing a marginal anisotropy in the out-of-plane direction. However, increasing the number of defects can distinguish anisotropy, enabling optimum spots of energy-saving for triggering mechanical actuation. The cubic-Snapology structure with a tetrahedron loop has isotropic stiffness, providing no optimum spots for triggering a transformation, as shown in Fig. 8c. The cubic-Snapology structures with disconnection constructing the planar square and spatial loops have lower directional stiffness. The lower directional stiffness implies that the Snapology structures can easily trigger transformation with low triggering mechanical energy than other modular origami topologies, as confirmed in Fig. 8d and Supplementary Video 3 in the SI.

Previously, Miura-origami^31,32^ and Snapology^33^ modular structures showed nonlinear folding behaviors during mechanical actuation due to self-locking, multi-stability, and other material nonlinearity. We expect similar behavior in our structures; as folding proceeds, the normal surface direction of weak spots for mechanical actuation can change for the global coordinate, coupled with the geometric, material, and kinematic nonlinearly, implying that our search for weak spots in Fig. 8 has a limitation for large deformation.

## Conclusion

We built building blocks of multi-DOF modular origami structures, including partial disconnection among modular units. By decomposing the motion of the core and tubular connection of the deformable modular units, we were able to determine the mobility of extended unit cells and network structures with the creation of loops by disconnection. Opposite to our intuition, an increase in disconnection rate does not necessarily reflect an increase in mobility, e.g., a network structure with spatial loops can maintain the same mobility (three DOF) as the fully filled structure while decreasing the mass by 25%. Our findings are valuable for the future design of soft modular robots, active architected materials, implanted modular medical devices, deployable structures, and active acoustic metamaterials. Our approach can expand the design space of multi-DOF modular origami structures by creating loops in spatial and planar directions with artificial defects—disconnection, providing tunable mobility and stiffness while minimizing mass. A defect is generally considered unacceptable mechanical damage in structural engineering. However, the defect presented in this work has the potential to offer increased control of the mobility of modular origami structures over perfectly filled ones, representing a paradigm shift in the design of robotic matter.

## Supplementary Information

Supplementary Information.

Supplementary Video S1.

Supplementary Video S2.

Supplementary Video S3.
