# Supplementary material for: Effect of disconnection of deformable units on the mobility and stiffness of 3D prismatic modular origami structures using angular kinematics
Source: Sci Rep. 2021 Sep 14;11:18259. doi: 10.1038/s41598-021-97609-5 (PMC8440563; doi:10.1038/s41598-021-97609-5)
Supplement: Supplementary file 1 — Supplementary Information. [file 41598_2021_97609_MOESM1_ESM.docx]

## **Supplementary Information**

**Effect of disconnection of deformable units on the mobility and stiffness of 3D prismatic modular origami structures using angular kinematics**

Kai Xiao^a^, Xiang Zhou^b^, Jaehyung Ju^a^

^a^ UM-SJTU Joint Institute, Shanghai Jiao Tong University, 800 Dongchuan Road, Shanghai, China

^b^ School of Aeronautic and Astronautic Engineering, Shanghai Jiao Tong University, 800 Dongchuan Road, Shanghai, China

# Fabrication of prototypes

We use a toy called Klikko^TM^ to make prototypes of our modular origami structures. Figure S1a shows the units - a rigid square lattice in light-blue and a rigid hinge in black, where the hinge connects the edges of two square lattices, as shown in Figure S1b. Due to the non-zero thickness of the square lattice, the hinge itself has a thickness so that the assembled lattices’ faces can fully contact each other when fully folded, as shown in Figure S1c. The assembly of four lattices with hinges behaves like a four-bar linkage, as shown in Figure S1d. The prototypes follow the same motion as those in [1] when the lattices are assembled to build a cubic Snapology structure, as shown in Figure S1e. Therefore, the prototypes demonstrate the motion of the analytical model of the modular origami in the main text.


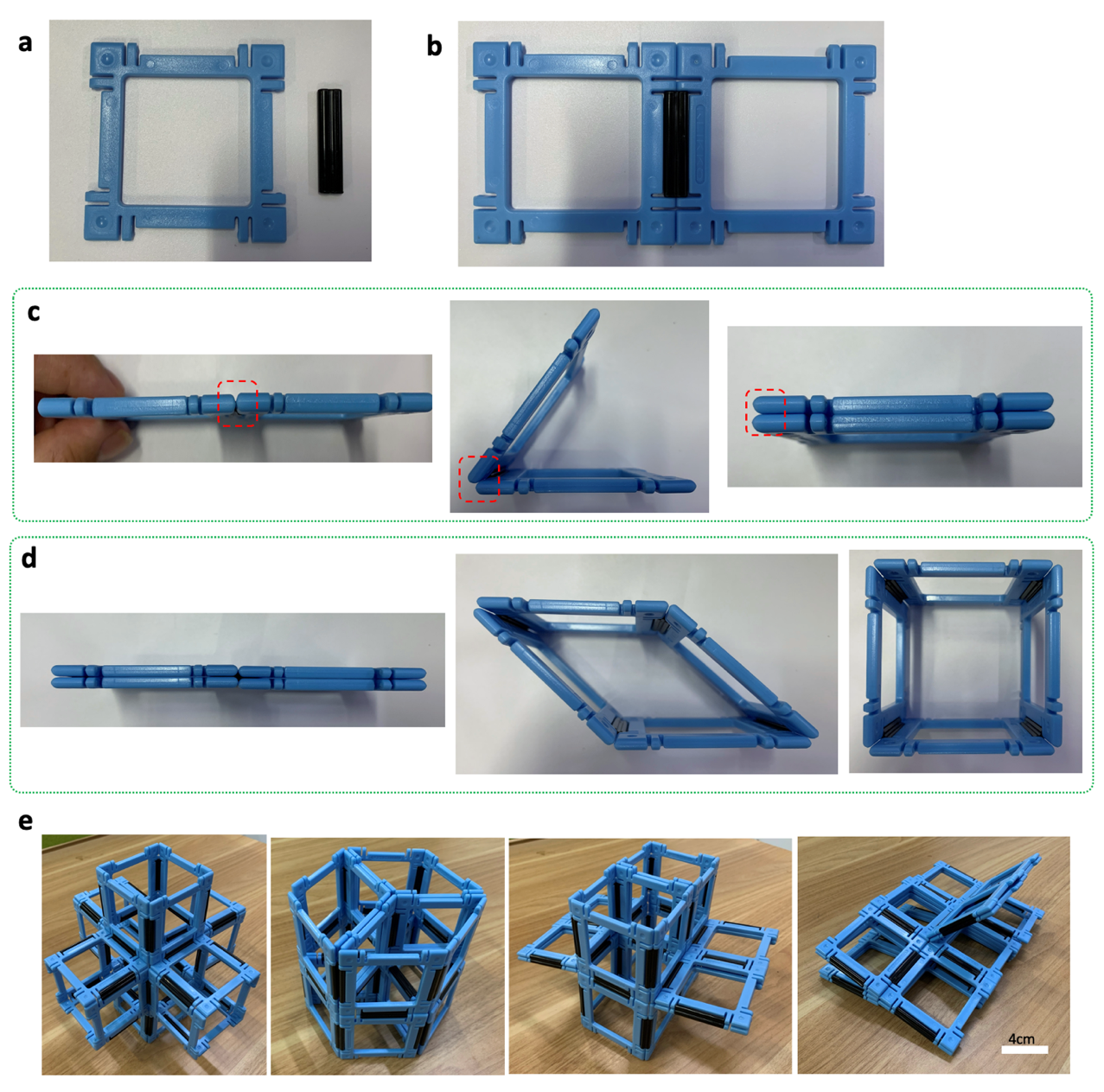


Figure S1. (a) Basic components of modular origami structures – a rigid square lattice in blud and a hinge in black; (b) an assembly of two square lattices with a hinge (top view); (c) folding motion of an assembly of two square lattices with a hinge (side view); (d) folding motion of an assembly of four-square lattices with four hinges (side view); (e) motion of an assembly of a cubic Snapology modular origami structure.

# Details of $\boldsymbol{\alpha}$ and $\boldsymbol{\beta}$ angles’ dependency in extended unit with two planar closed loops

The unit structure in Figure 5a consists of six irreducible unit cells with the original 18 DOF. The connecting tubes impose $\alpha_{2_{f}}=\alpha_{1_{g}}, \alpha_{2_{b}}=\alpha_{1_{c}}, \alpha_{2_{d}}=\alpha_{1_{a}},$ and

$$\alpha_{2_{g}}\boldsymbol{=}\alpha_{3_{c}}\boldsymbol{=}\mathrm{acos} \frac{\cos\beta_{2_{c}}(\cos\beta_{2_{c}}\cos\beta_{3_{c}}-\cos\alpha_{1_{c}}\sin\beta_{2_{c}}\sin\beta_{3_{c}})-\cos\beta_{3_{c}}}{\sin\beta_{2_{b}}\sin(\mathrm{acos}( \cos\beta_{2_{c}}\cos\beta_{3_{c}}-\cos\alpha_{1_{c}}\sin\beta_{2_{c}}\sin\beta_{3_{c}}))}, (S1)$$

$$\alpha_{1_{f}}=\alpha_{3_{b}}\boldsymbol{=}\mathrm{acos} \frac{\cos\beta_{1_{b}}(\cos\beta_{1_{b}}\cos\beta_{3_{b}}-\cos\alpha_{2_{b}}\sin\beta_{1_{b}}\sin\beta_{3_{b}})-\cos\beta_{3_{b}}}{\sin\beta_{1_{b}}\sin(\mathrm{acos}( \cos\beta_{1_{b}}\cos\beta_{3_{b}}-\cos\alpha_{2_{b}}\sin\beta_{1_{b}}\sin\beta_{3_{b}}))}, (S2)$$

$$\alpha_{2_{a}}=\alpha_{1_{b}}\boldsymbol{=}\mathrm{acos}\frac{(\cos\beta_{1_{b}}\cos\beta_{3_{b}}-\cos\alpha_{2_{b}}\sin\beta_{1_{b}}\sin\beta_{3_{b}})\cos\beta_{3_{b}}-\cos\beta_{1_{b}}}{\sin(\mathrm{acos} (\cos\beta_{1_{b}}\cos\beta_{3_{b}}-\cos\alpha_{2_{b}}\sin\beta_{1_{b}}\sin\beta_{3_{b}}))\sin\beta_{3_{b}}}, (S3)$$

$$\alpha_{1_{d}}=\alpha_{2_{c}}\boldsymbol{=}\mathrm{acos}\frac{(\cos\beta_{2_{c}}\cos\beta_{3_{c}}-\cos\alpha_{1_{c}}\sin\beta_{2_{c}}\sin\beta_{3_{c}})\cos\beta_{3_{c}}-\cos\beta_{2_{c}}}{\sin(\mathrm{acos} (\cos\beta_{2_{c}}\cos\beta_{3_{c}}-\cos\alpha_{1_{c}}\sin\beta_{2_{c}}\sin\beta_{3_{c}}))\sin\beta_{3_{c}}}, (S4)$$

# Derivation of mobility of the spatial closed-loop

The spatial closed-loop imposes six constraints from the tubular connections and another six constraints from the closed-loops on its original 18 mobility, leading 6 DOF $(6=18-6-6)$. After considering the tubular constraints, we have 12 DOF ($=6\times3-6$). These angles are $\beta_{3_{a}}, \alpha_{1_{a}},\beta_{3_{b}},\alpha_{2_{b}},\beta_{3_{f}},\alpha_{1_{f}},{\beta_{3}}_{g},\alpha_{2_{d}},\beta_{3_{d}},\alpha_{1_{h}},\beta_{3_{h}},$ and $\alpha_{2_{h}}$, as shown in Figure S2a.1. Therefore, we can simplify the structure in Figure S2a.1 into an equivalent spatial loop strip shown in Figure S2a.2 since this concise strip contains all position information of the extended unit. Next, the independent six loop constraints from this strip will be quantified.

Firstly, from the interior side of the spatial strip, we find:

$$\mathbf{v}_{\boldsymbol{ba}}\mathbf{+}\mathbf{v}_{\boldsymbol{fb}}\mathbf{+}\mathbf{v}_{\boldsymbol{gf}}\mathbf{-}\mathbf{v}_{\boldsymbol{da}}\boldsymbol{-}\mathbf{v}_{\boldsymbol{hd}}\boldsymbol{-}\mathbf{v}_{\boldsymbol{gh}}\boldsymbol{=0}(S5)$$

where these vectors are shown in Figure S2a.2. Equation S5 forces the internal nodes pointed by $\mathbf{v}_{\boldsymbol{ba}}+\mathbf{v}_{\boldsymbol{fb}}+\mathbf{v}_{\boldsymbol{gf}}$ to coincide with the node pointed by $\mathbf{v}_{\boldsymbol{da}}+\mathbf{v}_{\boldsymbol{hd}}+\mathbf{v}_{\boldsymbol{gh}}$. Since the vectors can be derived as complex functions of 11 angles, see the supplementary numerical code [2]. The coincident condition on interior nodes from Equation S5 imposes three constraints on the 11 DOF. For a brief description, we write the constraints from Equation S5 as

$$f_{1}\left( \beta_{3_{a}}, \alpha_{1_{a}},\beta_{3_{b}},\alpha_{2_{b}},\beta_{3_{f}},\alpha_{1_{f}},\alpha_{2_{d}},\beta_{3_{d}},\alpha_{1_{h}},\beta_{3_{h}},\alpha_{2_{h}} \right)=0,$$

$$f_{2}\left( \beta_{3_{a}}, \alpha_{1_{a}},\beta_{3_{b}},\alpha_{2_{b}},\beta_{3_{f}},\alpha_{1_{f}},\alpha_{2_{d}},\beta_{3_{d}},\alpha_{1_{h}},\beta_{3_{h}},\alpha_{2_{h}} \right)=0,$$

$f_{3}\left( \beta_{3_{a}}, \alpha_{1_{a}},\beta_{3_{b}},\alpha_{2_{b}},\beta_{3_{f}},\alpha_{1_{f}},\alpha_{2_{d}},\beta_{3_{d}},\alpha_{1_{h}},\beta_{3_{h}},\alpha_{2_{h}} \right)=0 (S6)$

In addition to constraints set in Equation S6, other constraints in the loop force the exterior nodes to coincide. For example, the vector $\mathbf{v}_{\beta_{3_{g}}}$ pointing to an exterior node (shown in Figure S2a.1) must coincide with vector ${\bar{\mathbf{v}}}_{{\beta_{3}}_{g}}$ since we can derive the motion of $\mathbf{v}_{\beta_{3_{g}}}$ from either the left hand side (LHS) branch as $\mathbf{v}_{\beta_{3_{g}}}=f_{\mathbf{v}_{\beta_{3_{g}}}}(\beta_{3_{a}}, \alpha_{1_{a}},\beta_{3_{b}},\alpha_{2_{b}},\beta_{3_{f}},\alpha_{1_{f}})$ or the right hand side (RHS) branch as ${\bar{\mathbf{v}}}_{{\beta_{3}}_{g}}=f_{{\bar{\mathbf{v}}}_{{\beta_{3}}_{g}}}(\alpha_{2_{d}},\beta_{3_{d}},\alpha_{1_{h}},\beta_{3_{h}},\alpha_{2_{h}})$.

The exterior coincidence condition for $\mathbf{v}_{\beta_{3_{g}}}$ and ${\bar{\mathbf{v}}}_{{\beta_{3}}_{g}}$ is:

$$\mathbf{v}_{\beta_{3_{g}}}-{\bar{\mathbf{v}}}_{{\beta_{3}}_{g}}=0 (S7)$$

The coincident condition EquationS7 must be satisfied, or the loop may lead to the wrong shape shown in Figure S2a.5.

Note that Equation S7 also give complex constraints on the 11 angles $\beta_{3_{a}}, \alpha_{1_{a}},\beta_{3_{b}},\alpha_{2_{b}},\beta_{3_{f}},\alpha_{1_{f}},\alpha_{2_{d}},\beta_{3_{d}},\alpha_{1_{h}},\beta_{3_{h}},\alpha_{2_{h}}$ And we write the constraints as:

$$f_{4}\left( \beta_{3_{a}}, \alpha_{1_{a}},\beta_{3_{b}},\alpha_{2_{b}},\beta_{3_{f}},\alpha_{1_{f}},\alpha_{2_{d}},\beta_{3_{d}},\alpha_{1_{h}},\beta_{3_{h}},\alpha_{2_{h}} \right)=0,$$

$$f_{5}\left( \beta_{3_{a}}, \alpha_{1_{a}},\beta_{3_{b}},\alpha_{2_{b}},\beta_{3_{f}},\alpha_{1_{f}},\alpha_{2_{d}},\beta_{3_{d}},\alpha_{1_{h}},\beta_{3_{h}},\alpha_{2_{h}} \right)=0,$$

$f_{6}\left( \beta_{3_{a}}, \alpha_{1_{a}},\beta_{3_{b}},\alpha_{2_{b}},\beta_{3_{f}},\alpha_{1_{f}},\alpha_{2_{d}},\beta_{3_{d}},\alpha_{1_{h}},\beta_{3_{h}},\alpha_{2_{h}} \right)=0 (S8)$

We note Equations S6 and S8 do not induce constraint on the angle $\beta_{3_{g}}$. This is because after establishing Equation S6 and S8,$\beta_{3_{g}}$ is a dependent variable on known vectors $\mathbf{v}_{gf}$ and $\mathbf{v}_{gh}$. However, we still can involve this angle into constrained variables by deriving $\mathbf{v}_{gh}$ in the LHS branch of the loop (Figure S2a.6) $\mathbf{v}_{gh}=f_{\mathbf{v}_{gh}}(\beta_{3_{a}}, \alpha_{1_{a}},\beta_{3_{b}},\alpha_{2_{b}},\beta_{3_{f}},\alpha_{1_{f}},\beta_{3_{g}} )$ and the RHS branch (Figure S2a.7) or ${\bar{\mathbf{v}}}_{gh}=f_{{\bar{\mathbf{v}}}_{gh}}(\alpha_{2_{d}},\beta_{3_{d}},\alpha_{1_{h}},\beta_{3_{h}})$.

Similar to the coincident condition in Equation S7, we have

$$\mathbf{v}_{gh}-{\bar{\mathbf{v}}}_{gh}=0 (S9)$$

Equation S9 leads

$$f_{7}\left( \beta_{3_{a}}, \alpha_{1_{a}},\beta_{3_{b}},\alpha_{2_{b}},\beta_{3_{f}},\alpha_{1_{f}},\alpha_{2_{d}},\beta_{3_{d}},\alpha_{1_{h}},\beta_{3_{h}},\alpha_{2_{h}},\beta_{3_{g}} \right)=0,$$

$$f_{8}\left( \beta_{3_{a}}, \alpha_{1_{a}},\beta_{3_{b}},\alpha_{2_{b}},\beta_{3_{f}},\alpha_{1_{f}},\alpha_{2_{d}},\beta_{3_{d}},\alpha_{1_{h}},\beta_{3_{h}},\alpha_{2_{h}},\beta_{3_{g}} \right)=0,$$

$f_{9}\left( \beta_{3_{a}}, \alpha_{1_{a}},\beta_{3_{b}},\alpha_{2_{b}},\beta_{3_{f}},\alpha_{1_{f}},\alpha_{2_{d}},\beta_{3_{d}},\alpha_{1_{h}},\beta_{3_{h}},\alpha_{2_{h}},\beta_{3_{g}} \right)=0 (S10)$

Now Equations S6, S8, and S10 give complete constraints set on 12 angles. However, it is hard to simplify the constraints from Equations S6 to S10 to find the independent ones. For doing so, we linearized these constraints as

$$\left[ \begin{aligned} \frac{\partial f_{1}}{\partial\beta_{3_{a}}} \frac{\partial f_{1}}{\partial\alpha_{1_{a}}} \ldots\frac{\partial f_{1}}{\partial\beta_{3_{g}}} \\ \frac{\partial f_{2}}{\partial\beta_{3_{a}}} \frac{\partial f_{2}}{\partial\alpha_{1_{a}}} \cdots\frac{\partial f_{2}}{\partial\beta_{3_{g}}} \\ \vdots\vdots\ddots\vdots\\ \frac{\partial f_{9}}{\partial\beta_{3_{a}}} \frac{\partial f_{9}}{\partial\alpha_{1_{a}}} \ldots\frac{\partial f_{9}}{\partial\beta_{3_{g}}} \end{aligned} \right]\cdot\left[ \begin{aligned} \begin{matrix} d\beta_{3_{a}} \\ d\alpha_{1_{a}} \\ \vdots\end{matrix} \\ d\beta_{3_{g}} \end{aligned} \right]=\boldsymbol{0 (}S11)$$

There are only six independent rows in Equation S11, which means there are six independent constraints on 12 angles (See the supplementary numerical code [2]). Therefore, we have found the number of independent constraints from the loop condition, plus the previous six tubular constraints. There are six independent angles on the spatial closed-loop structure. These six independent angles can be denoted as $\beta_{3_{a}}, \alpha_{1_{a}}, \beta_{3_{b}},\alpha_{1_{b}}, \beta_{3_{f}}, \alpha_{1_{f}}$, as denoted in Figure S2a.1. Some transformation state is shown in Figures S2b-S2g.


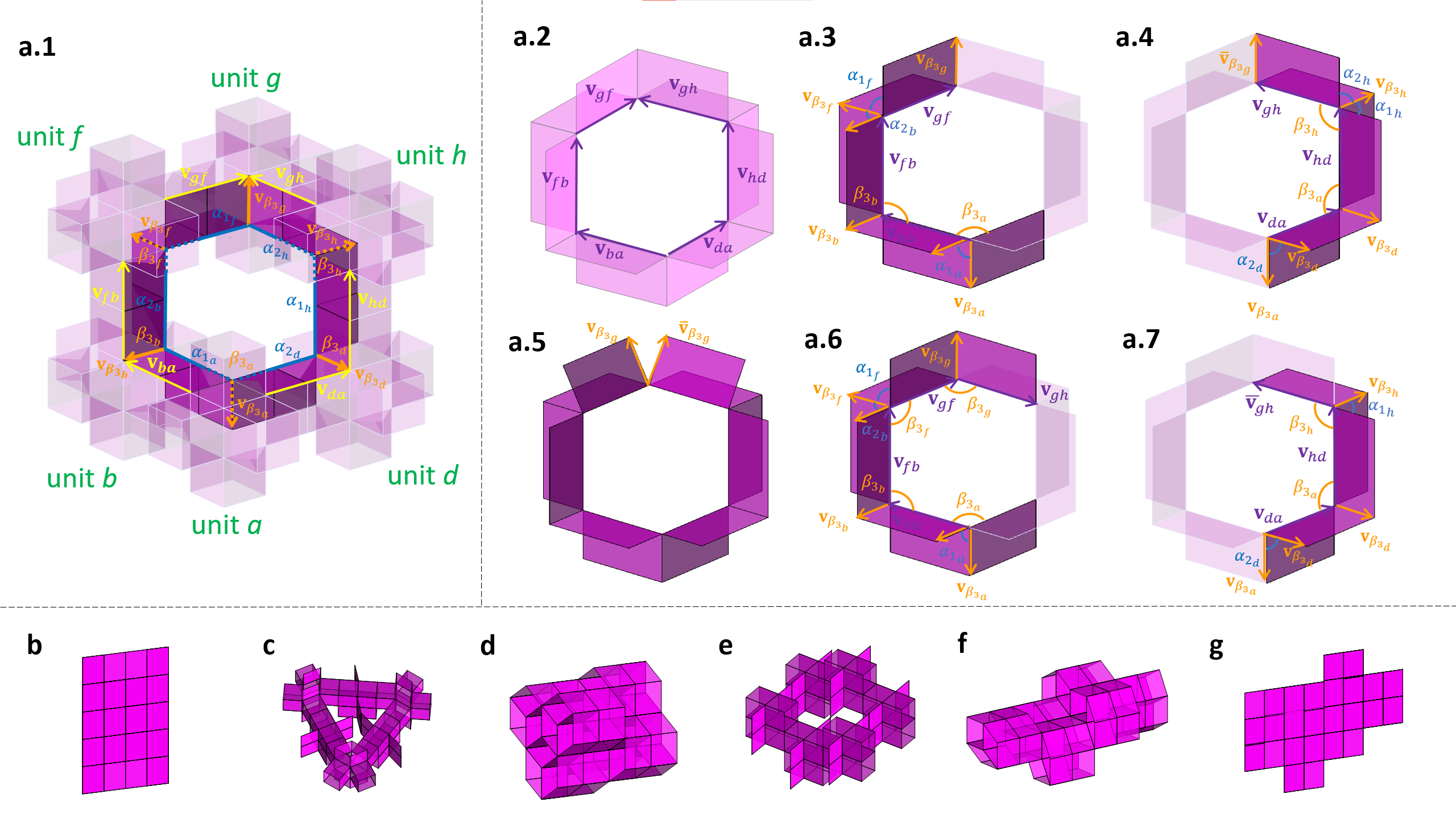


Figure S2. Spatial closed-loop cubic unit with 6 DOF. (a.1) Schematics of spatial closed loop structure’s selected independent angles $\alpha_{1a}, \alpha_{2b}, \alpha_{1f}, \beta_{3_{a}}, \beta_{3_{b}}, \beta_{3_{f}}$ Where $\alpha$ angles correspond to the rotational axis shown as the blue edge of the structure, and $\beta$ angles correspond to the orange one. Vectors $\boldsymbol{v}_{ba}, \boldsymbol{v}_{fb}, \boldsymbol{v}_{gf}, \boldsymbol{v}_{gh}, \boldsymbol{v}_{hd}, \boldsymbol{v}_{da},\boldsymbol{v}_{{\beta_{3}}_{a}}, \boldsymbol{v}_{{\beta_{3}}_{b}}, \boldsymbol{v}_{{\beta_{3}}_{f}}, \boldsymbol{v}_{{\beta_{3}}_{g}}, \boldsymbol{v}_{{\beta_{3}}_{h}}, \boldsymbol{v}_{{\beta_{3}}_{d}}$ shown in yellow and orange determine the whole motion of the structure. (a.2) spatial closed-loop condition on connected vectors. (a.3) Left-hand side of the spatial loop in light pink. (a.4) Right-hand side of the spatial loop in light pink. (a.5) wrong shape when the structure does not satisfy coincident condition (a.6) Left-hand-side of the spatial loop for involving angle ${\beta_{3}}_{g}$ (a.7) right-hand-side of the spatial loop for involving angle ${\beta_{3}}_{g}$ (b) A flat plate geometry generated by the spatial closed loop structure when set $\alpha_{1a}=0^{\circ}, \alpha_{2b}=0^{\circ},\alpha_{1f}=0^{\circ}, \beta_{3_{a}}, =0^{\circ}, \beta_{3_{b}}=0^{\circ}, \beta_{3_{f}}=0^{\circ},$ (c) Triangular connected geometry when set $\alpha_{1a}=90^{\circ}, \alpha_{2b}=90^{\circ},\alpha_{1f}=90^{\circ}, \beta_{3_{a}}=60^{\circ}, \beta_{3_{b}}=180^{\circ}, \beta_{3_{f}}=60^{\circ}.$ (d) The geometry with $\alpha_{1a}=60^{\circ}, \alpha_{2b}=60^{\circ},\alpha_{1f}=60^{\circ}, \beta_{3_{a}}=0^{\circ}, \beta_{3_{b}}=0^{\circ}, \beta_{3_{f}}=0^{\circ}.$ (e) The geometry with $\alpha_{1a}=90^{\circ}, \alpha_{2b}=90^{\circ},\alpha_{1f}=180^{\circ}, \beta_{3_{a}}=90^{\circ}, \beta_{3_{b}}=180^{\circ}, \beta_{3_{f}}=90^{\circ}.$ (f) The geometry with $\alpha_{1a}=120^{\circ}, \alpha_{2b}=60^{\circ},\alpha_{1f}=120^{\circ}, \beta_{3_{a}}=0^{\circ}, \beta_{3_{b}}=180^{\circ}, \beta_{3_{f}}=180^{\circ}.$ (g) The geometry with $\alpha_{1a}=0^{\circ}, \alpha_{2b}=0^{\circ},\alpha_{1f}=180^{\circ}, \beta_{3_{a}}=0^{\circ}, \beta_{3_{b}}=180^{\circ}, \beta_{3_{f}}=180^{\circ}.$

# Mobility analysis of network structures

This section provides a formulation procedure of mobility of network structures with selected extended unit cells.

**Case I: Planar network with fully filled extended unit cells**

Revisiting an extended unit cell $A$ $\left( n=4, c=1 \right)$ in Figure 2a of the main text, we know 5 DOF of the extended unit cell with the independent angles $\alpha_{1_{a}}$, $\alpha_{2_{a}}$, $\alpha_{1_{c}}$, $\alpha_{2_{c}},$and$\beta_{3_{a}}$ as obtained in Section III of the main text. Note that $n$ and $c$ are the number of cubic Snapology units and the number of closed-loops, as defined in the main text.

We search for the mobility of a network structure composed of the extended unit cells. For better visualization, we re-select the five independent angles ${\alpha_{1}}_{a}, {\alpha_{2}}_{c}, {\alpha_{2}}_{b},{\alpha_{2}}_{a},$and ${\beta_{3}}_{c}$ of the extended unit cell $A$, as shown in Figure S3a. For better notation to distinguish the independent angles for multiple extended unit cells, we rename the five independent angles of the extended unit cell $A$ as ${\alpha_{1}}_{A}, {\alpha_{2}}_{A}, {\alpha_{3}}_{A},{\alpha_{4}}_{A},$and $\beta_{A}$, while addressing an adjacent extended unit cell $B$ whose independent angles are ${\alpha_{1}}_{B}, {\alpha_{2}}_{B}, {\alpha_{3}}_{B},{\alpha_{4}}_{B}$and $\beta_{B}$, as shown in Figure S3b. Observing the motion of the two extended unit cells, we find the following three kinematic constraints between $A$ and $B$,

$${\alpha_{1}}_{A}={\alpha_{1}}_{B}, {\alpha_{3}}_{A}={\alpha_{3}}_{B}, \beta_{A}=\beta_{B} (S12)$$

Therefore, we have seven $\left( =10-3 \right)$ independent angles for a network structure with $A$ and $B$: ${\alpha_{1}}_{A}, {\alpha_{2}}_{A}, {\alpha_{3}}_{A},{\alpha_{4}}_{A},{\alpha_{1}}_{B},{\alpha_{2}}_{B}, \beta_{A}$. Adding another extended unit cell $C$, as shown in Figure S3c, we impose the following three kinematic constraints:

${\alpha_{4}}_{A}={\alpha_{4}}_{C}, {\alpha_{2}}_{A}={\alpha_{2}}_{C}, \beta_{A}=\beta_{C} (S13)$

So far, there are nine $\left( =3\times5-2\times3 \right)$ independent angles for the extended unit cells $A$, $B$, and $C$: e.g., ${\alpha_{1}}_{A}, {\alpha_{2}}_{A}, {\alpha_{3}}_{A},{\alpha_{4}}_{A},{\alpha_{1}}_{B},{\alpha_{2}}_{B},{\alpha_{1}}_{C},{\alpha_{3}}_{C},$ and $\beta_{A}$.

Finally, adding an additional extended unit cell $D$, as shown in Figure S3d, we have those five kinematic constraints:

$${\alpha_{1}}_{C}={\alpha_{1}}_{D}, {\alpha_{2}}_{B}={\alpha_{2}}_{D},{\alpha_{3}}_{C}={\alpha_{3}}_{D},{\alpha_{4}}_{B}={\alpha_{4}}_{D}, \beta_{A}=\beta_{D} (S14)$$

The five constraints in Equation S14 imply that the extended unit cell $D$ does not affect the overall mobility. Therefore, the fully filled planar network with four extended unit cells has nine DOF with nine independent angles: ${\alpha_{1}}_{A}, {\alpha_{2}}_{A}, {\alpha_{3}}_{A},{\alpha_{4}}_{A},{\alpha_{1}}_{B},{\alpha_{2}}_{B},{\alpha_{1}}_{C},{\alpha_{3}}_{C}, \beta_{A}$.

We can construct a general mobility equation of an $i\times j$ network structure where $i$ and $j$ are positive integers, e.g., $i,j=1,2, 3, 4, 5, \ldots, N.$ As illustrated in Figure S3e, we divide a network structure into four regions: the unit region $(i=1,j=1)$, the $i$-region $(j=1,i>1)$, the $j$-region $(j>1,i=1)$, and the $ij$-region $(j>1,i>1)$. Notably, the four regions independently affect the overall DOF. The unit region has 5 DOF, which is the mobility of the extended unit cell. The $i$ and $j$ regions bring $2(i-1)$ and $2(j-1)$ DOFs, respectively. Because the $ij$ region does not affect the overall DOF of a network structure, the mobility of a network structure with $i\times j$ extended unit cells is

$${DOF}_{n=4, c=1}^{P}=2i+2j+1 (S15)$$

where the superscript $P$ denotes a planar tessellation.


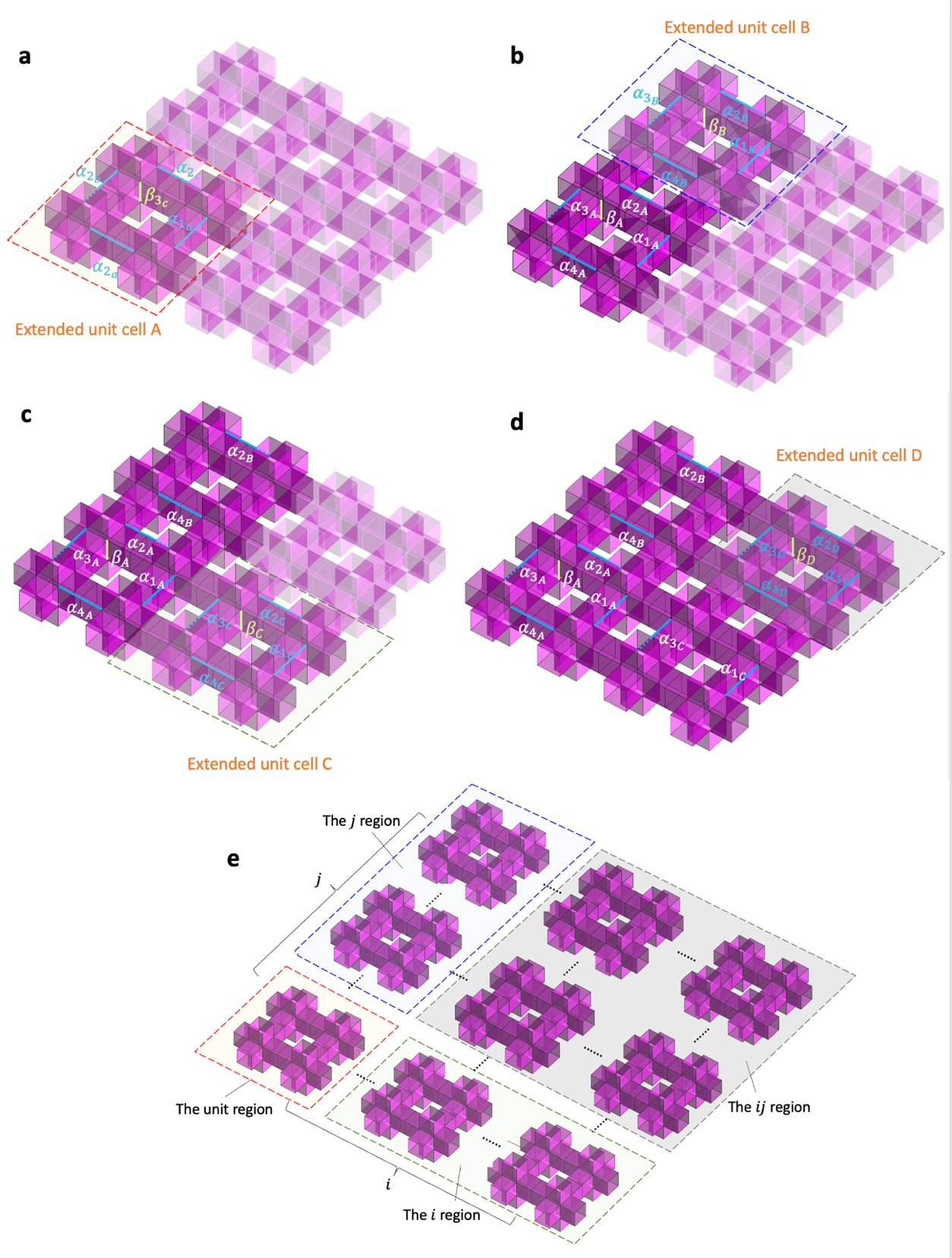


Figure S3. Procedure to obtain mobility of network structures consisting of extended unit cells $\left( n=4, c=1 \right)$; (a) extended unit cell A; (b) a network structure with two extended unit cells A and B; (c) a network structure with three extended unit cells A, B, and C; (d) a network structure with four extended unit cells A, B, C, and D; (e) four regions for the generalized analysis of a network structure with $3\times3$ extended unit cells.

**Case II: Planar network with partially filled extended unit cells**

We analyse a network structure made of partially filled extended unit cell $\left( n=3, c=0 \right)$ in Figure 2c of the main text. Knowing seven DOFs of the extended unit cell $A$ with independent angles - ${\alpha_{1}}_{A}, {\alpha_{2}}_{A}, {\alpha_{3}}_{A},{\alpha_{4}}_{A}$, $\beta_{1_{A}},\beta_{2_{A}},$and $\beta_{3_{A}}$ in Section III of the main text, we add extended unit cells $B$ and $C$ in the $i$ and $j$ rows, as illustrated in Figures S4a and S4b. The added $B$ and $C$ impose one kinematic constraint for each row: ${\alpha_{4}}_{A}={\alpha_{4}}_{C}$ and ${\alpha_{3}}_{A}={\alpha_{3}}_{B}$ for the $i$ and $j$ rows, respectively, producing a general mobility formula of the network structure with $A$, $B$, and $C$ as $7+6(j-1)+6(i-1)$.

An extended unit cell $D$ on the $ij$ region makes an internal loop with eight cubic units, as illustrated in Figure S4c. While making the loop, $D$ coupled with the motion of $A$, $B$, and $C$. As indicated in Figure S4c, the motion of the loop can be decoupled to two out-of-plane modes and one in-plane mode. Out-of-plane mode #1 imposes

$${\alpha_{3}}_{C}={\alpha_{3}}_{D}, {\alpha_{4}}_{B}={\alpha_{4}}_{D} (S16)$$

Out-of-plane mode #2 is associated with those four angles: ${\alpha_{2}}_{A}, {\alpha_{1}}_{A}, {\alpha_{1}}_{B}$ and ${\alpha_{3}}_{C}$. However, only three of them are independent due to this constraint:

$$f_{1}\left( {\alpha_{2}}_{A}, {\alpha_{1}}_{A}, {\alpha_{1}}_{B},{\alpha_{3}}_{C} \right)=0, (S17)$$

The in-plane mode function like an eight-bar linkage with revolute joints; only five out of eight angles $\beta_{3_{A}}, \beta_{1_{A}}, \beta_{3_{C}},\beta_{2_{C}},\beta_{3_{D}},\beta_{1_{B}},\beta_{3_{B}}$, and $\beta_{2_{A}}$ remain independent [3] due to the following three kinematic constraints:

$$f_{2}\left( \beta_{3_{A}}, \beta_{1_{A}}, \beta_{3_{C}},\beta_{2_{C}},\beta_{3_{D}},\beta_{1_{B}},\beta_{3_{B}}, \beta_{2_{A}} \right)=0,$$

$$f_{3}\left( \beta_{3_{A}}, \beta_{1_{A}}, \beta_{3_{C}},\beta_{2_{C}},\beta_{3_{D}},\beta_{1_{B}},\beta_{3_{B}}, \beta_{2_{A}} \right)=0,$$

$$f_{4}\left( \beta_{3_{A}}, \beta_{1_{A}}, \beta_{3_{C}},\beta_{2_{C}},\beta_{3_{D}},\beta_{1_{B}},\beta_{3_{B}}, \beta_{2_{A}} \right)=0, (S18)$$

Notably, adding D increases only one DOF $\left( =7-6 \right)$ due to the six constraints in Equations S16-S18. Therefore, the generalized DOF, including the unit, $i$, $j$, and $ij$ regions, is $7+6\left( j-1 \right)+6\left( i-1 \right)+1\times\left( i-1 \right)\times\left( j-1 \right)$. The complete form is

$${DOF}_{n=3,c=0}^{P}=ij+5\left( i+j \right)-4 (S19)$$

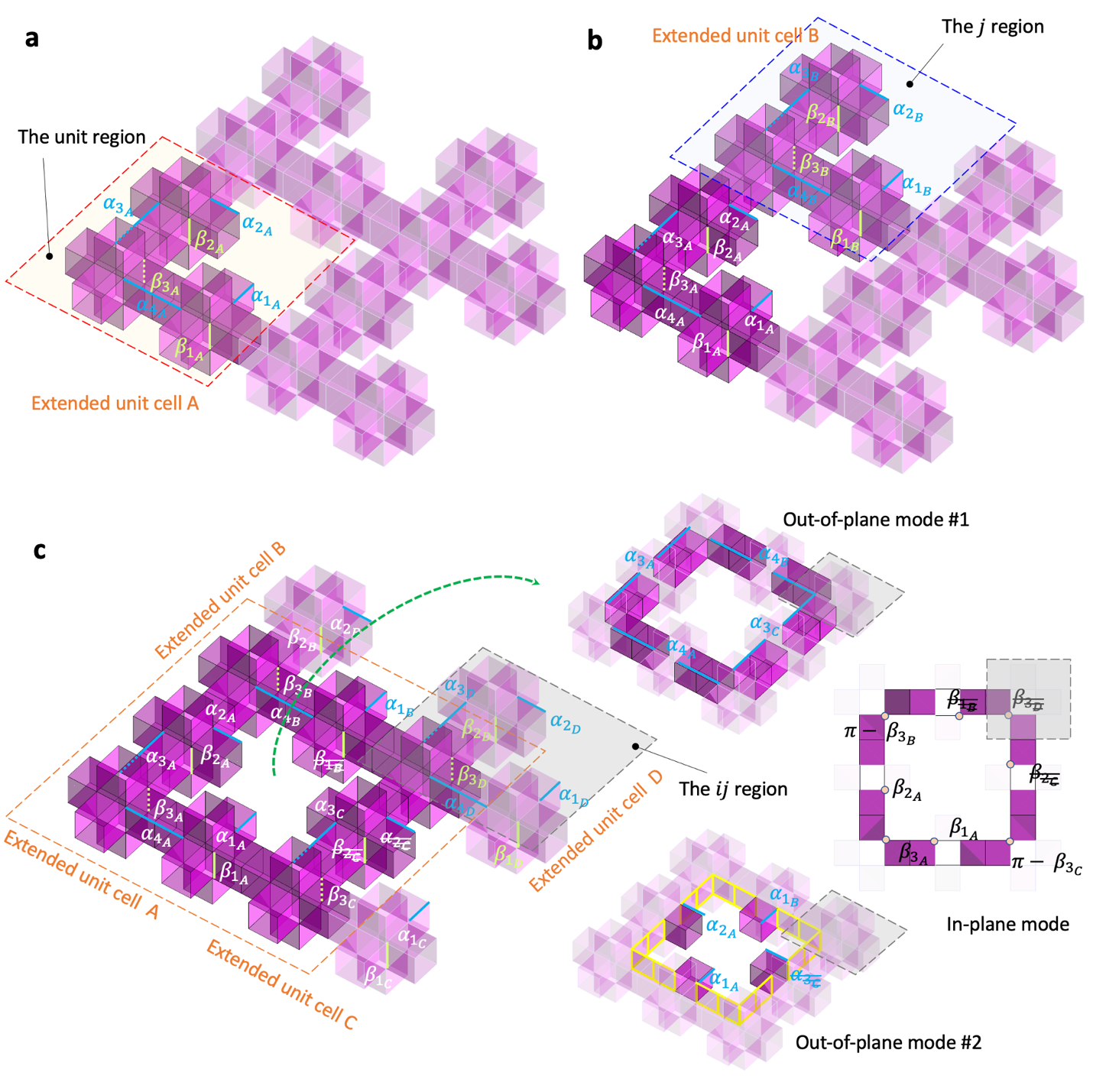


Figure S4. Procedure to obtain mobility of network structures consisting of extended unit cells$\left( n=3, c=0 \right)$; (a) extended unit cell A; (b) a network structure with two extended unit cells A and B; (c) a network structure with three extended unit cells A, B, C, and D. The new loop creates two out-of-plane modes and one in-plane mode. Note that we applied a strikethrough to the dependent angles.

**Case III: Spatial network with partially filled extended unit cells**

Adding the $k$ regions on vertical stacking to the planar motion, we can formulate the mobility of spatial network structures. As shown in Figure S5, we divide with following eight regions: the unit region $(i=1,j=1,k=1)$, the $i$-region $(j=1,k=1,i>1)$, the $j$-region $(k=1,i=1,j>1)$, the $k$-region $(i=1,j=1,k>1)$, the $ij$-region $(k=1,j>1,i>1)$, the $jk$-region $(i=1,j>1,k>1)$, the $ik$-region $(j=1,i>1,k>1)$ and the $ijk$-region $(i>1,j>1,k>1)$.

We choose an extended unit cell $A$ $\left( n=4, c=0 \right)$ shown in Figure 4a of the main text. Knowing that the extended unit cell $A$ has nine DOF with independent angles $\alpha_{1_{A}},\alpha_{2_{A}},\alpha_{3_{A}}, \alpha_{4_{A}}, {\beta_{1}}_{A},{\beta_{2}}_{A},{\beta_{3}}_{A},{\beta_{8}}_{A},$ and ${\beta_{9}}_{A}$, we start adding an extended unit cell $B$ on the $i$-region as shown in Figure S5c, producing one constraint:

$$\alpha_{4_{A}}=\alpha_{4_{B}} (S20)$$

Therefore, adding one extended unit cell on the $i$-region has eight DOF $=\left( 9-1 \right)$. The added mobility with the $i-1$ extended unit cells is $8(i-1)$.

Similarly, as shown in Figure S5d, adding an extended unit cell $C$ on the $j$-region leads to one constraint:

$$\alpha_{3_{A}}=\alpha_{3_{C}} (S21)$$

Similar to the planar closed loop in the previous analysis with Equations S16-S18, adding one extended unit cell to the $ij$region create a new planar loop as shown in Figure S5e, leading to the following constraints on the out-of-plane mode #1:

$$\alpha_{4_{C}}=\alpha_{4_{D}}, \alpha_{3_{B}}=\alpha_{3_{D}} (S22)$$

There are three constraints on the in-plane mode:

$$f_{1}\left( \beta_{3_{A}}, \beta_{1_{A}}, \beta_{3_{B}},\beta_{2_{A}},\beta_{2_{B}},\beta_{3_{C}},\beta_{1_{C}}, \beta_{3_{D}} \right)=0,$$

$$f_{2}\left( \beta_{3_{A}}, \beta_{1_{A}}, \beta_{3_{B}},\beta_{2_{A}},\beta_{2_{B}},\beta_{3_{C}},\beta_{1_{C}}, \beta_{3_{D}} \right)=0,$$

$$f_{3}\left( \beta_{3_{A}}, \beta_{1_{A}}, \beta_{3_{B}},\beta_{2_{A}},\beta_{2_{B}},\beta_{3_{C}},\beta_{1_{C}}, \beta_{3_{D}} \right)=0, (S23)$$

There is a constraint on the out-of-plane mode #2:

$$f_{4}\left( {\alpha_{1}}_{A}, {\alpha_{2}}_{A}, {\alpha_{2}}_{B},{\alpha_{1}}_{C} \right)=0, (S24)$$

With the six constraints from Equations S22-S24, adding one extended unit cell on the $ij$region produce thee DOFs $\left( =9-6 \right)$, implying an increase of DOF by $3(i-1)(j-1)$ for the $(i-1)(j-1)$ extended unit cells on the $ij$ region.

Now, the constraint with the extended unit cell along the vertical direction $k$ in Figure S5f produces the following relation:

$$\alpha_{7_{A}}=\alpha_{7_{E}} (S25)$$

Adding extended unit cell $F$ on the $ik$region, as shown in Figure S5g, create a new planar loop, leading to the following constraints:

$$\alpha_{4_{F}}=\alpha_{4_{E}}, \alpha_{7_{F}}=\alpha_{7_{B}} (S26)$$

$$f_{5}\left( \beta_{7_{A}}, \beta_{4_{A}}, \beta_{7_{B}},\beta_{8_{A}},\beta_{8_{B}},\beta_{7_{E}},\beta_{4_{E}}, \beta_{8_{F}} \right)=0,$$

$$f_{6}\left( \beta_{7_{A}}, \beta_{4_{A}}, \beta_{7_{B}},\beta_{8_{A}},\beta_{8_{B}},\beta_{7_{E}},\beta_{4_{E}}, \beta_{8_{F}} \right)=0,$$

$$f_{7}\left( \beta_{7_{A}}, \beta_{4_{A}}, \beta_{7_{B}},\beta_{8_{A}},\beta_{8_{B}},\beta_{7_{E}},\beta_{4_{E}}, \beta_{8_{F}} \right)=0, (S27)$$

And

$$f_{8}\left( {\alpha_{8}}_{A}, {\alpha_{5}}_{A}, {\alpha_{5}}_{E},{\alpha_{8}}_{B} \right)=0, (S28)$$

Similarly, adding extended unit cells $G$ on the $jk$ region also creates a new planar loop, producing the following constraints:

$$\alpha_{3_{E}}=\alpha_{3_{G}}, \alpha_{7_{G}}=\alpha_{7_{C}} (S29)$$

$$f_{9}\left( \beta_{6_{A}}, \beta_{5_{A}}, \beta_{6_{C}},\beta_{9_{A}},\beta_{9_{C}},\beta_{6_{E}},\beta_{5_{E}}, \beta_{6_{G}} \right)=0,$$

$$f_{10}\left( \beta_{6_{A}}, \beta_{5_{A}}, \beta_{6_{C}},\beta_{9_{A}},\beta_{9_{C}},\beta_{6_{E}},\beta_{5_{E}}, \beta_{6_{G}} \right)=0,$$

$$f_{11}\left( \beta_{6_{A}}, \beta_{5_{A}}, \beta_{6_{C}},\beta_{9_{A}},\beta_{9_{C}},\beta_{6_{E}},\beta_{5_{E}}, \beta_{6_{G}} \right)=0, (S30)$$

And

$$f_{12}\left( {\alpha_{6}}_{A}, {\alpha_{9}}_{A}, {\alpha_{6}}_{E},{\alpha_{9}}_{C} \right)=0, (S31)$$

So far, we find that adding $i-1$, $j-1,$and $k-1$ extended unit cells on the $i,j$, and $k$ regions, respectively, produces $8\left[ \left( i-1 \right)+\left( j-1 \right)+\left( k-1 \right) \right]$ DOFs for each region. Adding extended unit cells on the $ij,jk,$ and $ik$ regions increases their mobility by $3\left[ \left( i-1 \right)(j-1)+\left( i-1 \right)(j-1)+\left( i-1 \right)(k-1) \right]$ for each region.

Finally, we add an extended unit cell $H$ on the $ijk$region in Figure S5h, creating three planar closed-loops with the following 15 constraints as shown in Figure S5i:

$$\alpha_{3_{F}}=\alpha_{3_{H}}, \alpha_{4_{G}}=\alpha_{4_{H}} (S32)$$

$$f_{13}\left( \beta_{3_{E}}, \beta_{1_{E}}, \beta_{3_{F}},\beta_{2_{E}},\beta_{3_{G}},\beta_{3_{G}},\beta_{2_{F}}, \beta_{3_{H}} \right)=0,$$

$$f_{14}\left( \beta_{3_{E}}, \beta_{1_{E}}, \beta_{3_{F}},\beta_{2_{E}},\beta_{3_{G}},\beta_{3_{G}},\beta_{2_{F}}, \beta_{3_{H}} \right)=0,$$

$$f_{15}\left( \beta_{3_{E}}, \beta_{1_{E}}, \beta_{3_{F}},\beta_{2_{E}},\beta_{3_{G}},\beta_{3_{G}},\beta_{2_{F}}, \beta_{3_{H}} \right)=0, (S33)$$

$$f_{16}\left( {\alpha_{1}}_{E}, {\alpha_{2}}_{E}, {\alpha_{2}}_{F},{\alpha_{1}}_{G} \right)=0, (S34)$$

and

$$\alpha_{7_{D}}=\alpha_{7_{H}} (S35)$$

$$f_{17}\left( \beta_{6_{B}}, \beta_{5_{B}}, \beta_{6_{D}},\beta_{9_{B}},\beta_{9_{D}},\beta_{6_{F}},\beta_{5_{F}}, \beta_{6_{H}} \right)=0,$$

$$f_{18}\left( \beta_{6_{B}}, \beta_{5_{B}}, \beta_{6_{D}},\beta_{9_{B}},\beta_{9_{D}},\beta_{6_{F}},\beta_{5_{F}}, \beta_{6_{H}} \right)=0,$$

$$f_{19}\left( \beta_{6_{B}}, \beta_{5_{B}}, \beta_{6_{D}},\beta_{9_{B}},\beta_{9_{D}},\beta_{6_{F}},\beta_{5_{F}}, \beta_{6_{H}} \right)=0, \left( S36 \right)$$

$$f_{20}\left( {\alpha_{6}}_{B}, {\alpha_{9}}_{B}, {\alpha_{9}}_{D},{\alpha_{6}}_{F} \right)=0, (S37)$$

as well as

$$f_{21}\left( \beta_{7_{C}}, \beta_{4_{C}}, \beta_{7_{D}},\beta_{8_{C}},\beta_{8_{D}},\beta_{7_{G}},\beta_{4_{G}}, \beta_{7_{H}} \right)=0,$$

$$f_{22}\left( \beta_{7_{C}}, \beta_{4_{C}}, \beta_{7_{D}},\beta_{8_{C}},\beta_{8_{D}},\beta_{7_{G}},\beta_{4_{G}}, \beta_{7_{H}} \right)=0,$$

$$f_{23}\left( \beta_{7_{C}}, \beta_{4_{C}}, \beta_{7_{D}},\beta_{8_{C}},\beta_{8_{D}},\beta_{7_{G}},\beta_{4_{G}}, \beta_{7_{H}} \right)=0, \left( S38 \right)$$

$$f_{24}\left( {\alpha_{8}}_{C}, {\alpha_{5}}_{C}, {\alpha_{8}}_{D},{\alpha_{5}}_{G} \right)=0, (S39)$$

However, only nine constraints from Equations S32-S39 are independent, implying no change of DOF $\left( =9-9 \right)$ with an added extended unit cell $H$.

Therefore, the network structure with the $2\times2\times2$ extended unit cells has a total of 30 kinematic constraints, providing 42 DOFs $\left( =8\times9-30 \right)$ with independent angles: ${\alpha_{4}}_{A},{\alpha_{3}}_{A},$ ${\beta_{3}}_{A},$ ${\alpha_{1}}_{A}, {\beta_{1}}_{A},$ ${\beta_{3}}_{B},$ ${\alpha_{3}}_{B},$ ${\alpha_{1}}_{C},$ $\alpha_{4_{C}},$ $\beta_{3_{C}},$ $\beta_{2_{A}},$ $\alpha_{2_{A}},$ $\beta_{8_{A}},$ $\beta_{1_{E}},$ $\alpha_{4_{E}},$ $\beta_{9_{A}},$ $\beta_{6_{E}},$ $\alpha_{3_{E}},$ ${\beta_{1}}_{B},$ ${\alpha_{1}}_{B},$ ${\beta_{1}}_{D}, {\alpha_{1}}_{D}, {\beta_{1}}_{F}, {\alpha_{1}}_{F},$ ${\beta_{1}}_{H}, {\alpha_{1}}_{H},$ $\alpha_{2_{C}},$ $\beta_{2_{C}},$ $\alpha_{2_{D}}, \beta_{2_{D}}, \alpha_{2_{G}},\beta_{2_{G}},\alpha_{2_{H}},\beta_{2_{H}},\beta_{8_{F}},\beta_{9_{F}},\beta_{8_{E}},$ $\beta_{9_{E}},\beta_{8_{G}},\beta_{9_{G}},\beta_{8_{H}},$ and $\beta_{9_{H}}.$

The mobility equation of an $i\times j\times k$ network structure with the extended unit cell is $9+8\left[ \left( i-1 \right)+\left( j-1 \right)+(k-1) \right]+3\left[ \left( i-1 \right)(j-1)+\left( j-1 \right)(k-1)+\left( k-1 \right)(i-1) \right]$, further simplified as:

$${DOF}_{n=4, c=0}^{S}=2\left( i+j+k \right)+3\left( ij+jk+ik \right)-6 (S40)$$

where the subscript $S$ denotes a spatial tessellation.


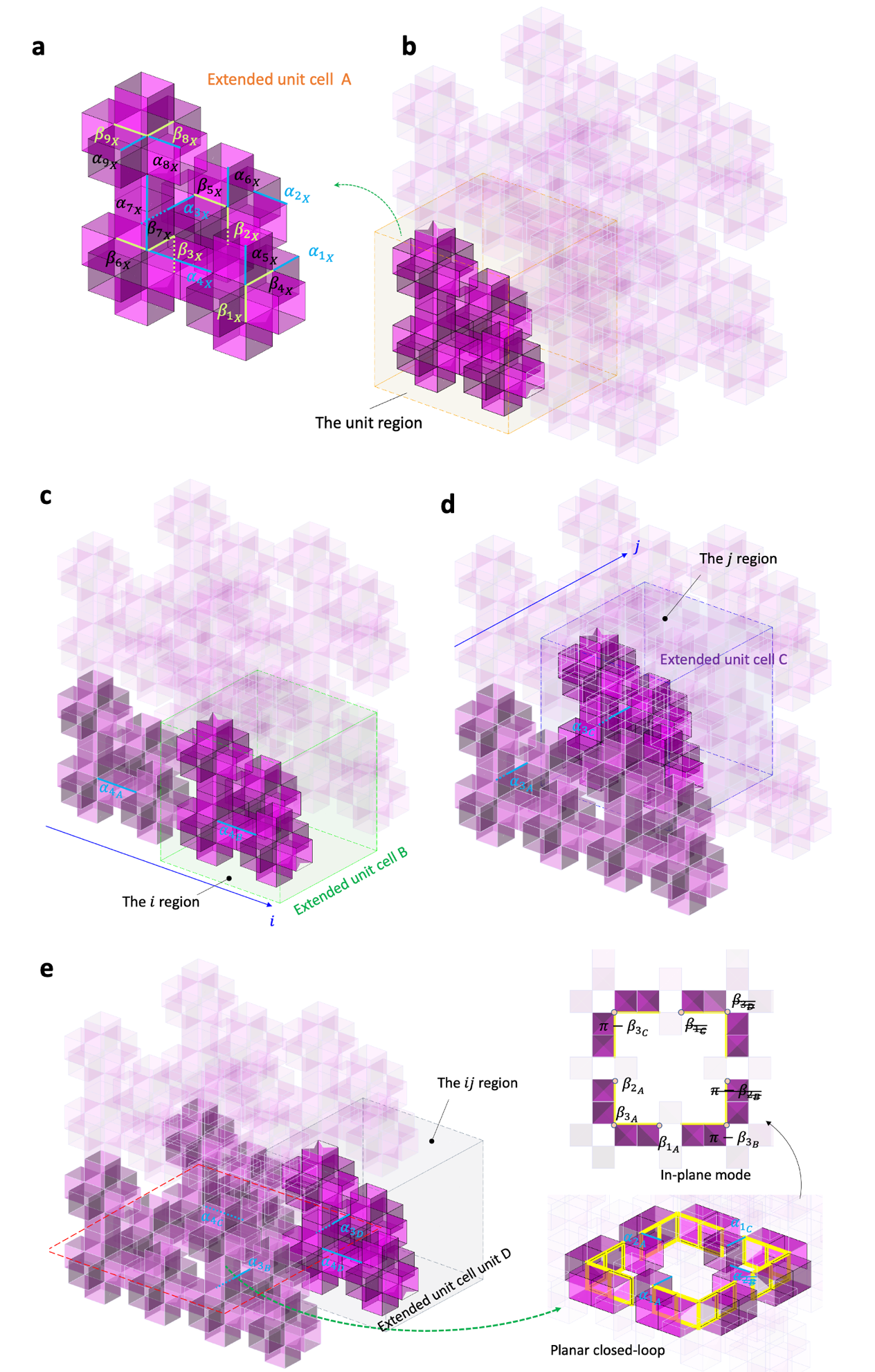


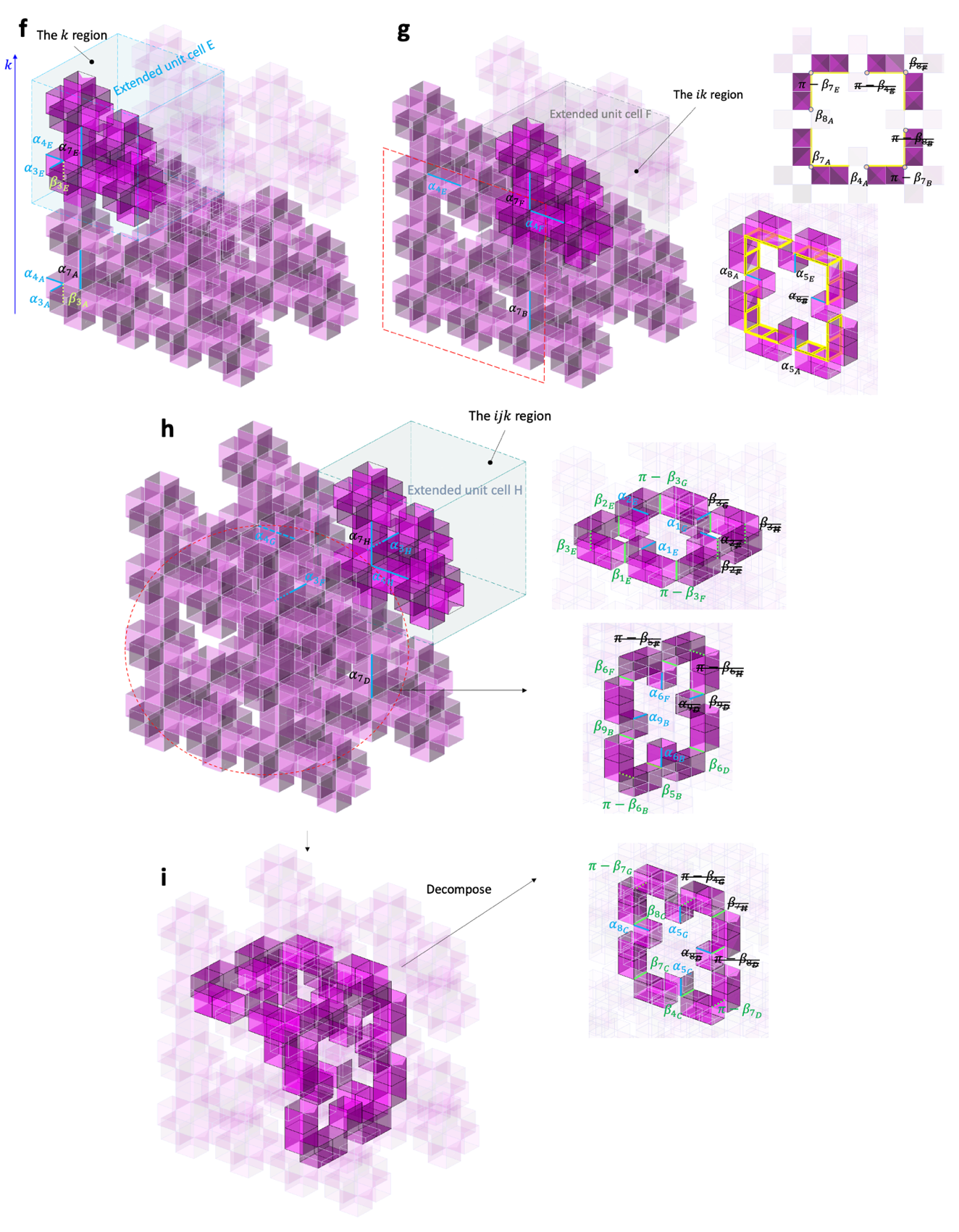


Figure S5. Procedure to obtain mobility of network structures consisting of extended unit cells $\left( n=4, c=0 \right)$; (a) extended unit cell A with independent angles $\alpha_{1_{A}},\alpha_{2_{A}},\alpha_{3_{A}}, \alpha_{4_{A}}, {\beta_{1}}_{A},{\beta_{2}}_{A},{\beta_{3}}_{A},{\beta_{8}}_{A},{\beta_{9}}_{A}$; (b) the extended unit cell A on the unit region of a $2\times2\times2$ network structure; (c) addition of an extended unit cell B along the direction of $i$; (d) addition of an extended unit cell C along the $j$ direction; (e) addition of an extended unit cell D on the $ij$region forming a planar closed-loop; (f) addition of an extended unit cell E along the k direction; (g) addition of an extended unit cell F on the ik region; (h) addition of an extended unit cell H on the ijk region; (i) decomposition of the formed closed-loops. Note that we applied a strikethrough to the dependent angles.

# Directional stiffness of the modular origami structures

We adapt the method from [4] to model the deformable face of origami structures with the elastic hinge. We assume the hinges have linearly rotational stiffness; the bending of the face is captured by one bar with rotational, linear stiffness placed on the diagonal of the face, the elongation of the face is measured by four linear elastic bars on each face’s perimeter. Facet shearing is captured by two bars placed on the diagonal of the face (see Figure S6) [4,5,6].


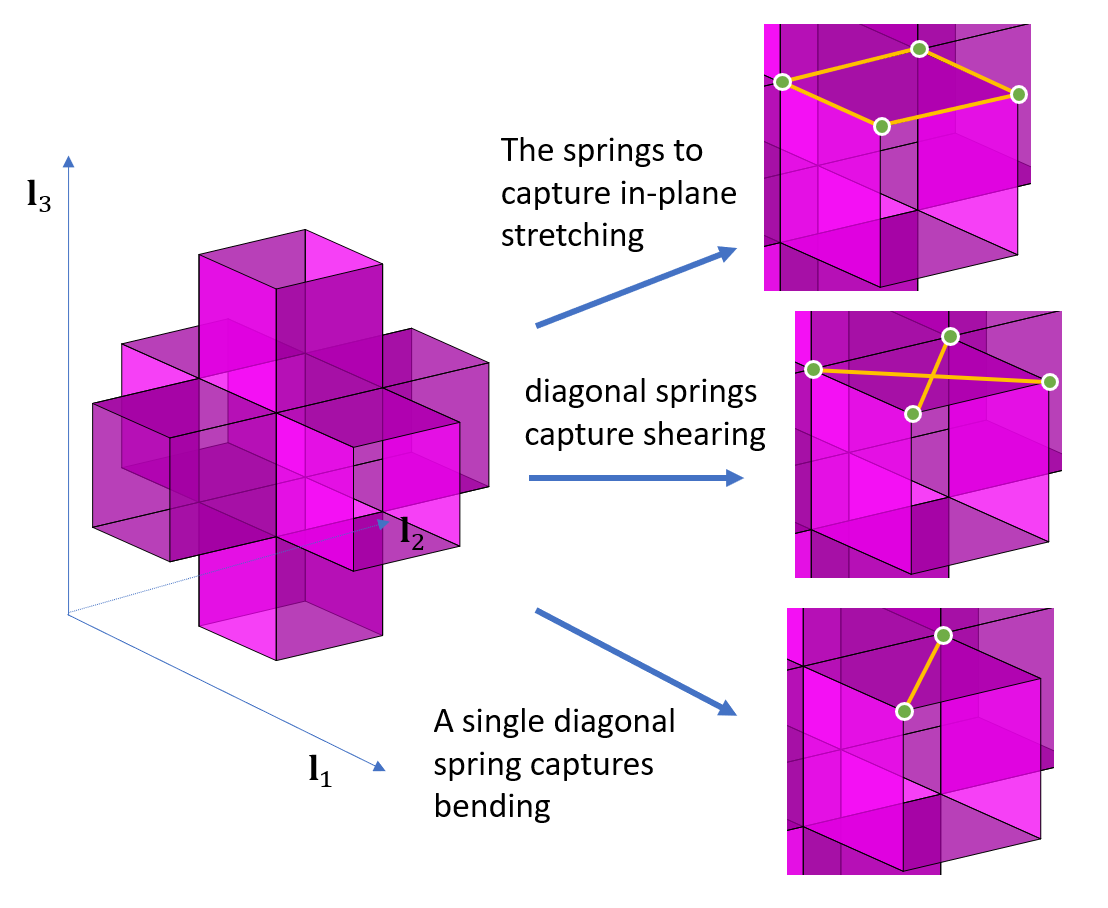


Figure S6. Schematics of bar model on origami structures.

***Strain Energy***

After building the bar model, strain energy $W_{strain}$ of such structures can be written as:

$W_{strain}=W_{hinge}+W_{bending}+W_{elongation}+W_{shearing} (S41)$

$W_{hinge}$ can be expressed as for an origami structure with a total number of hinges, $H$:

$$W_{hinge}=\frac{1}{2}d\boldsymbol{\theta}^{T}\mathbf{E}_{h}d\boldsymbol{\theta(}S42\mathbf{)}$$

where $d\boldsymbol{\theta}$ is $\left[ {d\theta}_{1}, {d\theta}_{2}, \ldots,d\theta_{H} \right]^{T}$ the infinitesimal change of the angle of the hinge. $\mathbf{E}_{h}=\mathrm{diag}\left( E_{h_{1}}, E_{h_{2}},\ldots,E_{h_{H}} \right),$ and $E_{h_{r}}$ is the $r$-th stiffness of hinges.

Since $d\boldsymbol{\theta}$ is a function of vertices’ displacement $d\mathbf{x}$ [4], we rewrite Equation S42 as

$$W_{hinge}=\frac{1}{2}d\mathbf{x}^{T}\mathbf{J}_{h}^{T}\mathbf{E}_{h}\mathbf{J}_{h}d\mathbf{x (}S43\mathbf{)}$$

where $d\boldsymbol{\theta}=\mathbf{J}_{h}\cdot d\mathbf{v}\boldsymbol{,}$ $\mathbf{J}_{h}$ is a jacobian matrix.

Similarly, we write $W_{bending}$ as

$$W_{bending}=\frac{1}{2}d\mathbf{x}^{T}\mathbf{J}_{b}^{T}\mathbf{E}_{b}\mathbf{J}_{b}d\mathbf{x (}S44\mathbf{)}$$

where$\mathbf{E}_{b}=\mathrm{diag}\left( E_{b_{1}}, E_{b_{2}},\ldots,E_{b_{B}} \right)$, and $E_{b_{r}}$ is the rotational stiffness of the $r$-th bar for capturing bending deformation.

The strain energy for elongation $W_{elongation}$ is represented by

$$W_{elongation}=\frac{1}{2}d\mathbf{e}^{T}\mathbf{E}_{e}d\mathbf{e (}S45\mathbf{)}$$

where $d\mathbf{e}$ is $\left[ {de}_{1}, {de}_{2}, \ldots,de_{E} \right]^{T}$, $de_{i}$ is the $i$-th elongating bar’s length change. $\mathbf{E}_{e}=\mathrm{diag}\left( E_{e_{1}}, E_{e_{2}},\ldots,E_{e_{E}} \right),$ $E$ is the total number of such bars. Since $d\mathbf{e=}\mathbf{J}_{e}d\mathbf{x}$, where $\mathbf{J}_{e}$ is the Jacobean between $d\mathbf{x}$ and $d\mathbf{e}$, we rewrite $W_{elongation}$as

$$W_{elongation}=\frac{1}{2}d\mathbf{x}^{T}\mathbf{J}_{e}^{T}\mathbf{E}_{e}\mathbf{J}_{e}d\mathbf{x (}S46\mathbf{)}$$

Similarly, we write the strain energy for shear:

$$W_{shearing}=\frac{1}{2}d\mathbf{x}^{T}\mathbf{J}_{sh}^{T}\mathbf{E}_{sh}\mathbf{J}_{sh}d\mathbf{x (}S47\mathbf{)}$$

***Bar stiffness***

We assume the base material of the structure has Young's modulus of $E_{s}$ and Poisson’s ratio of $\nu=\frac{1}{3}$. Besides, the thickness of faces $t=0.01\cdot L$, where $L$ is the length of the face’s edge. Then the stiffness $E_{h}, E_{b}, E_{e}, E_{sh}$can be further calculated as [4,6]

$$E_{h}=C_{b}\frac{E_{s}Lt^{3}}{24\left( 1-\nu^{2} \right)}\left( \frac{1}{t} \right)^{\frac{1}{3}} (S48)$$

$$E_{b}=C_{b}\frac{E_{s}t^{3}}{12\left( 1-\nu^{2} \right)}\left( \frac{L}{t} \right)^{\frac{1}{3}} (S49)$$

$$E_{e}=\frac{E_{s}t}{2(1+v)} (S50)$$

$$E_{sh}=\frac{\sqrt{2}E_{s}t\nu}{1-v^{2}} (S51)$$

where $C_{b}is$ a stiffness coefficient, in which we apply 0.441 for $C_{b}$ since we only consider small displacements [6].

***Directional loading***

We apply the directional loading and the corresponding displacement with a periodic boundary condition. The applied periodic boundary condition is:

$$d\mathbf{x}_{a}\boldsymbol{-}{d\mathbf{x}}_{b}\boldsymbol{=}\sum_{i=1}^{3} \sigma_{i}\mathbf{G}\mathbf{l}_{i}\boldsymbol{(}S52\boldsymbol{)}$$

where $d\mathbf{x}_{a}$ and ${d\mathbf{x}}_{b}$ are the displacements of periodically located vertices’ pair denoted by index $a$ and $b$. The second-order displacement gradient tensor $\mathbf{G}$ has a matrix form: $\mathbf{G=}\left[ \begin{matrix} G_{11} & G_{12} & G_{13} \\ G_{21} & G_{22} & G_{23} \\ G_{31} & G_{32} & G_{33} \end{matrix} \right]$**.** $\mathbf{l}_{i}\boldsymbol{(}i=1,2,3\boldsymbol{)}$ are the three lattice vectors of the unit and $\sigma_{i}\in\{-1,0,1\}$.

For enforcing the constraints from Equation S52, we reform $\mathbf{G}$ into a vector form $\mathbf{g}=\left[ G_{11}\boldsymbol{,}G_{12}\mathbf{,}G_{13}\mathbf{,}G_{21}\mathbf{,}G_{22}\boldsymbol{,\ldots,}G_{33} \right]^{T}$, and building a matrix $\mathbf{C}$ to enforce constraints for $d\mathbf{x}$ and $\mathbf{g}$:

$$\left[ \begin{matrix} d\mathbf{x} \\ \mathbf{g} \end{matrix} \right]\boldsymbol{=}\boldsymbol{C\cdot s} \left( S53 \right)$$

Where the vector $\mathbf{s}$is the small independent displacements from $d\mathbf{x}$ and $\mathbf{g}$.

We substitute Equation S53 into Equations S43, S44, S46, and S47, which leads

$$W_{hinge}=\frac{1}{2}\mathbf{s}^{T}\mathbf{C}^{T} \left[ \mathbf{J}_{h}\mathbf{0} \right]^{T} \mathbf{E}_{h}\left[ \mathbf{J}_{h}\mathbf{0} \right]\mathbf{Cs} (S54)$$

$$W_{bending}=\frac{1}{2}\mathbf{s}^{T}\mathbf{C}^{T} \left[ \mathbf{J}_{b}\mathbf{0} \right]^{T} \mathbf{E}_{b}\left[ \mathbf{J}_{b}\mathbf{0} \right]\mathbf{Cs} (S55)$$

$$W_{elongation}=\frac{1}{2}\mathbf{s}^{T}\mathbf{C}^{T} \left[ \mathbf{J}_{e}\mathbf{0} \right]^{T} \mathbf{E}_{e}\left[ \mathbf{J}_{e}\mathbf{0} \right]\mathbf{Cs} (S56)$$

$$W_{shearing}=\frac{1}{2}\mathbf{s}^{T}\mathbf{C}^{T} \left[ \mathbf{J}_{sh}\mathbf{0} \right]^{T} \mathbf{E}_{sh}\left[ \mathbf{J}_{sh}\mathbf{0} \right]\mathbf{Cs} (S57)$$

Using the principle of minimum potential energy:

$$\frac{\partial W_{potential}}{\partial\mathbf{s}}=0 (S58)$$

where $W_{potential}=W_{strain}-T$. Note $T=\mathbf{g}^{T}\mathbf{f}$ is the work done by external forces $\mathbf{f}$, where $\mathbf{f=}\left[ f_{11}\boldsymbol{,}f_{12}\mathbf{,}f_{13}\mathbf{,}f_{21}\mathbf{,}f_{22}\boldsymbol{,\ldots,}f_{33} \right]^{T}$. Equation S58 leads

$$\mathbf{C}^{T}\left( \left[ \mathbf{J}_{h}\mathbf{0} \right]^{T} \mathbf{E}_{h}\left[ \mathbf{J}_{h}\mathbf{0} \right]+\left[ \mathbf{J}_{b}\mathbf{0} \right]^{T} \mathbf{E}_{b}\left[ \mathbf{J}_{b}\mathbf{0} \right]+ \left[ \mathbf{J}_{h}\mathbf{0} \right]^{T} \mathbf{E}_{h}\left[ \mathbf{J}_{h}\mathbf{0} \right]+\left[ \mathbf{J}_{sh}\mathbf{0} \right]^{T} \mathbf{E}_{sh}\left[ \mathbf{J}_{sh}\mathbf{0} \right] \right)\mathbf{C}d\mathbf{s=}\mathbf{C}^{T}\left[ \begin{matrix} \boldsymbol{0} \\ \mathbf{f} \end{matrix} \right] (S59)$$

Furthermore, we fix a node while applying a uniaxial loading by letting $G_{11}\neq0$ in Equation S53, leaving $G_{22}$ and $G_{33}$free and $G_{ij}=0$for $i\neq j$ as the boundary condition. The directional loading in space can be determined by rotating the unit with two loading directions with angles $\theta$ and $\gamma$ in Figure 7 of the main text. Assuming the unit has macroscopic initial volume $V$, the magnitude of stiffness for varying $\theta$ and $\gamma$ is

$E^{*}=\frac{f_{11}}{V\cdot G_{11}} (S60)$

Furthermore, the above calculation is implemented in MATLAB, and an open-source code is provided on GitHub [2].

# References:

1. Overvelde, J. T., De Jong, T. A., Shevchenko, Y., Becerra, S. A., Whitesides, G. M., Weaver, J. C., Hoberman, C. & Bertoldi, K., A three-dimensional actuated origami-inspired transformable metamaterial with multiple degrees of freedom, *Nature communications,* **7**, 1-8 (2016).
2. MATLAB codes – computation of kinematic constraints, directional stiffness, and numerical computation of mobility of network structures with defects, [SI-code-for-Effect-of-disconnected-Snapology- (github.com)](https://github.com/KaiXiao55/SI-code-for-Effect-of-disconnected-Snapology-) <https://github.com/KaiXiao55/SI-code-for-Effect-of-disconnected-Snapology->
3. You, Z. & Chen, Y. Motion structures: deployable structural assemblies of mechanisms, *CRC Press*, 2011.
4. Overvelde, J. T., Weaver, J. C., Hoberman, C. & Bertoldi, K. Rational design of reconfigurable prismatic architected materials. *Nature,* **541**, 347-352 (2017).
5. Schenk, M. & Guest, S. D. Geometry of Miura-folded metamaterials. *Proceedings of the National Academy of Sciences,* **110**, 3276-3281 (2013).
6. Filipov, E. T., Tachi, T. & Paulino, G. H. Origami tubes assembled into stiff, yet reconfigurable structures and metamaterials. *Proceedings of the National Academy of Sciences,* **112**, 12321-12326 (2015).

# List of supplementary videos:

S1. Transformation of a cubic Snapology unit with three independent angles – $\alpha_{1}$, $\alpha_{2}$, and $\beta_{3}$. [link](https://youtu.be/XmprT7AvxOA)

S2. Transformation of an extended unit cell $\left( n=2\times2 \right)$. [link](https://youtu.be/fnxt4suoiNE)

S3. Comparison of energy consumption for directional mechanical actuation with a small displacement. [link](https://www.youtube.com/watch?v=tMuZH2wc_j0)
